# EpiMOLAS: an intuitive web-based framework for genome-wide DNA methylation analysis

**DOI:** 10.1186/s12864-019-6404-8

**Published:** 2020-04-02

**Authors:** Sheng-Yao Su, I-Hsuan Lu, Wen-Chih Cheng, Wei-Chun Chung, Pao-Yang Chen, Jan-Ming Ho, Shu-Hwa Chen, Chung-Yen Lin

**Affiliations:** 1 Taiwan International Graduate Program (TIGP) on Bioinformatics, Academia Sinica, Taipei, Taiwan; 2 Institute of Information Science, Academia Sinica, Taipei, Taiwan; 3 Institute of Biomedical Informatics, National Yang-Ming University, Taipei, Taiwan; 4 Institute of Population Health Sciences, National Health Research Institutes, Zhunan Miaoli, Taiwan; 5 Institute of Plant and Microbial Biology, Academia Sinica, Taipei, Taiwan; 6 TMU Research Center of Cancer Translational Medicine, Taipei Medical University, Taipei, Taiwan; 7 Institute of Fisheries Science, College of Life Science, National Taiwan University, Taipei, Taiwan

**Keywords:** WGBS pipeline, Docker, Galaxy platform, DNA methylation data analysis

## Abstract

**Background:**

DNA methylation is a crucial epigenomic mechanism in various biological processes. Using whole-genome bisulfite sequencing (WGBS) technology, methylated cytosine sites can be revealed at the single nucleotide level. However, the WGBS data analysis process is usually complicated and challenging.

**Results:**

To alleviate the associated difficulties, we integrated the WGBS data processing steps and downstream analysis into a two-phase approach. First, we set up the required tools in Galaxy and developed workflows to calculate the methylation level from raw WGBS data and generate a methylation status summary, the *mtable*. This computation environment is wrapped into the Docker container image *DocMethyl*, which allows users to rapidly deploy an executable environment without tedious software installation and library dependency problems. Next, the *mtable* files were uploaded to the web server *EpiMOLAS_web* to link with the gene annotation databases that enable rapid data retrieval and analyses.

**Conclusion:**

To our knowledge, the EpiMOLAS framework, consisting of *DocMethyl* and *EpiMOLAS_web*, is the first approach to include containerization technology and a web-based system for WGBS data analysis from raw data processing to downstream analysis. EpiMOLAS will help users cope with their WGBS data and also conduct reproducible analyses of publicly available data, thereby gaining insights into the mechanisms underlying complex biological phenomenon. The Galaxy Docker image *DocMethyl* is available at https://hub.docker.com/r/lsbnb/docmethyl/.

*EpiMOLAS_web* is publicly accessible at http://symbiosis.iis.sinica.edu.tw/epimolas/.

## Background

DNA methylation on cytosine is an epigenetic modification that occurs in numerous biological processes, including transposable element silencing, mammalian gene regulation, genomic imprinting, and X chromosome inactivation [1]. Compared to other epigenetic modifications, cytosine methylation is a relatively stable epigenetic mark inherited during cell divisions. In vertebrates, methylated cytosines were first identified in gene promoters as well as the transcribed regions. Besides those found in promoters, the methylation pattern in intragenic transcribed gene body regions are also evolutionarily conserved among organisms, and the control and biological mechanisms remain to be explored [2].

Over the past decades, several protocols and assays, such as MBD-seq [3], MeDIP-seq [4], reduced representation bisulfite sequencing (RRBS) [5], whole-genome bisulfite sequencing (WGBS) [6], and Infinium Methylation 450 K/EPIC array [7], have been developed to profile genome-wide DNA methylation. MBD-seq and MeDIP-seq techniques are affinity enrichment-based methods that use antibodies to extract the methylated genomic regions. They are cost-effective approaches but have a potentially confounding bias in varying CpG density. Several pipelines are available and used for these type of datasets [8–11].

Bisulfite sequencing (BS-seq) has become a popular technology to analyze DNA methylation. It is based on the differential chemical reactions to bisulfite treatment between unmethylated and methylated cytosines. The treatment of sodium bisulfite converts unmethylated cytosines (C) to uracils (U) and uracils (U) to thymines (T) after PCR amplification [12]. Coupled with next-generation sequencing technology and downstream bioinformatics strategies, the bisulfite-converted reads are mapped using a wild-card or three-letter mapping strategy [13], after which, the percentage of cells that are methylated at each genomic cytosine site can be estimated. Recent advances in sequencing technology make it possible to identify the methylation states on a genome-wide scale at single-base resolution and allow WGBS data to be more accessible. Here, we focused our analysis on WGBS data. Although many approaches and tools are available to handle WGBS data [13–15], there is still a lack of automated workflows and well-annotated databases with customizable downstream analyses for the users’ own datasets.

## Implementation

By leveraging the Linux container virtualization technology (LXC) and the community-supported Galaxy platform [16], we developed a seamless and ready-to-use workflow, which can be rapidly deployed and executed on a single machine or a distributed cloud computing environment, avoiding tedious software installation and library dependency problems. This Galaxy Docker container, *DocMethyl*, includes FastQC [17], Trim Galore [18], Bismark [19], the in-house program *EpiMolas.jar* [20], and two built-in workflows to streamline each processing step, including (1) clean-up, to trim the adapter sequence and low-quality bases from raw reads; (2) read mapping, to align trimmed reads to the reference genome; (3) methylation calling, to extract the methylation status of each cytosine throughout the genome; and (4) methylatn scoring, to calculate the methylation level of each gene.

**Fig. 1:**
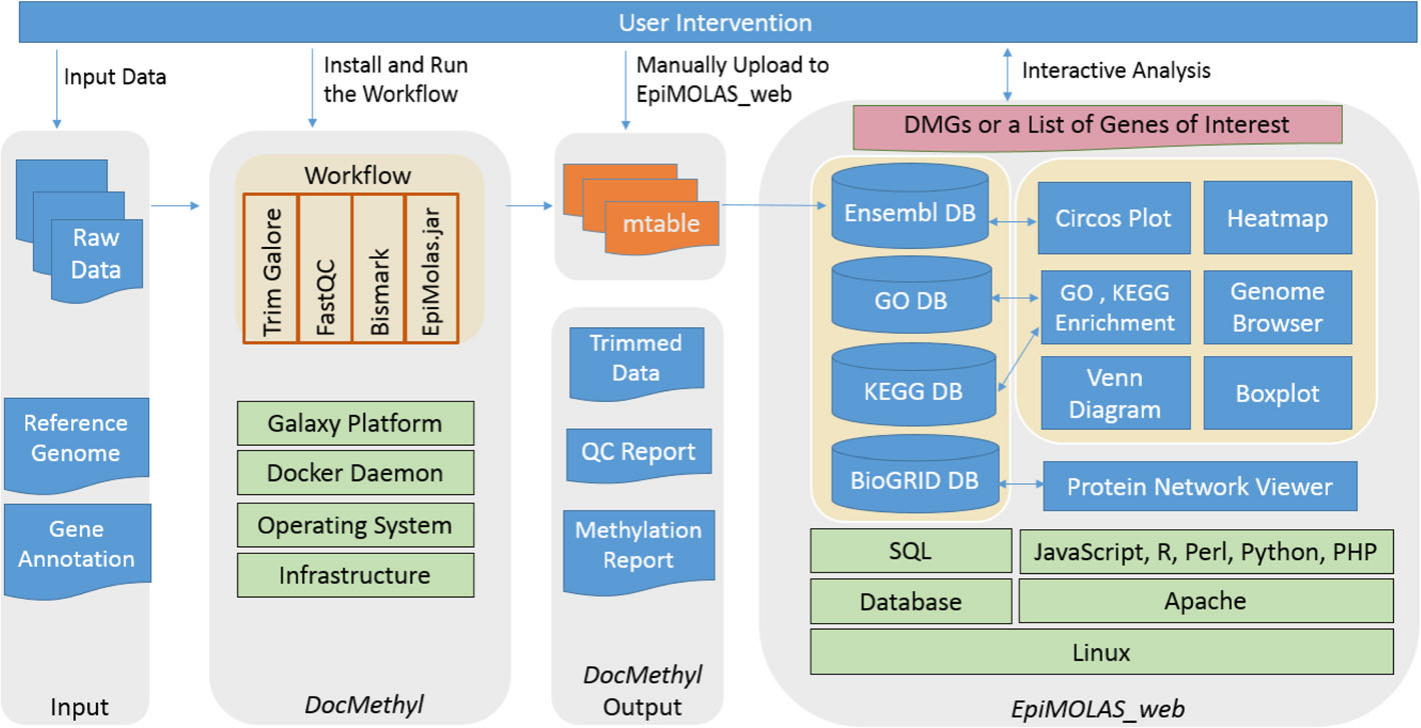
Implementation and integration of *DocMethyl* and *EpiMOLAS_web*

*EpiMOLAS_web* is an online platform that connects the *DocMethyl* summary file, *mtable*, to a rich collection of annotations of the human genome (GRCh37, GRCh38), the mouse genome (GRCm38), and the Arabidopsis genome (TAIR10) [21]. We implemented the system using the Ubuntu 14.04, Apache 2.04, PostgreSQL 9.1, PHP 5.1, and Bootstrap responsive web design (RWD) framework to create a user-friendly interface. Data integration and score calculation in the *EpiMOLAS_web* analysis process are implemented in Java, Python, and PHP. Moreover, to help users understand their results, several data visualization tools are integrated into the system. For example, we combined Biodalliance (version 0.13.7) [22], Clustergrammer [23] and Circos (version 0.69) [24] allowing the users to browse the coordination of genes on the chromosomes and display the gene-based DNA methylation profiles. In the enrichment analysis, the gene function annotations, including Gene Ontology (GO) terms [25] and Kyoto Encyclopedia of Genes and Genomes (KEGG) pathway database [26], were collected in Feb. 2019. We also utilized the BioGRID protein interaction data (version 3.5.170) [27] and implemented a dedicated protein interaction network viewer in JavaScript, Cytoscape.js [28], HTML5, and MySQL for the topological data analyses and visualization. The integrated architecture of *DocMethyl* and *EpiMOLAS_web* is depicted in Fig. 1.

## Results

**Fig. 2:**
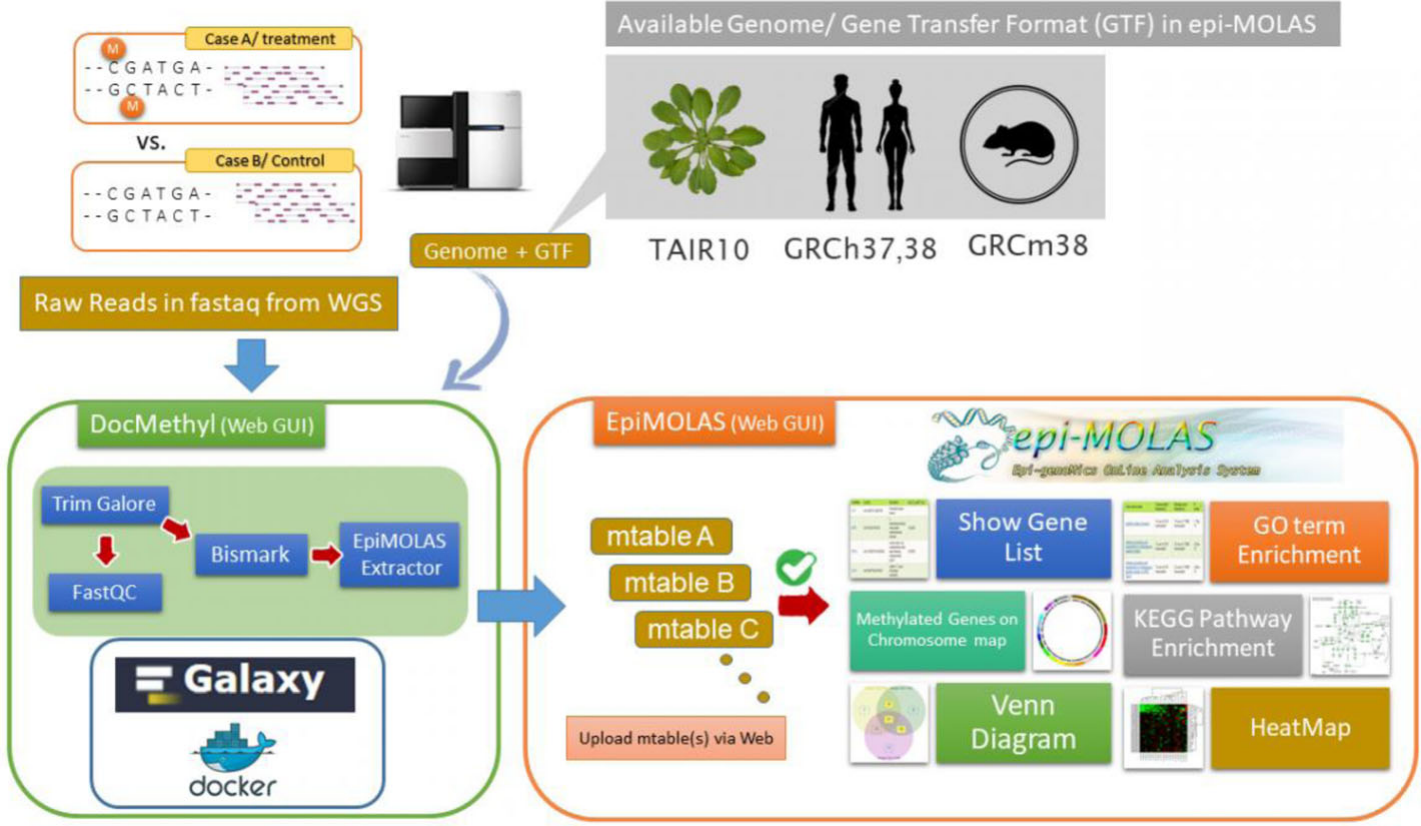
EpiMOLAS, including *DocMethyl* and *EpiMOLAS_web*, is a two-phase approach for WGBS data analysis. *DocMethyl* requires Bisulfite-Seq read data, reference genome sequences, and gene annotation files to determine the DNA methylation status. The resulting *mtable* files are uploaded to the *EpiMOLAS_web* server via the web user interfaces for users to perform various types of data analyses and visualization modules

To alleviate the burden of BS-seq data processing and analysis, we developed **EpiMOLAS**, a two-phase approach which consists of *DocMethyl* and *EpiMOLAS_web*. The Docker container, *DocMethyl*, completes the intensive short reads processing tasks and generates a tab-delimited methylation summary file (namely *mtable*) for each WGBS dataset. The online web server, *EpiMOLAS_web*, links the output *mtable* files with gene annotation databases and provides versatile downstream analyses, as shown in Fig. 2.

### DocMethyl

**Figure 3:**
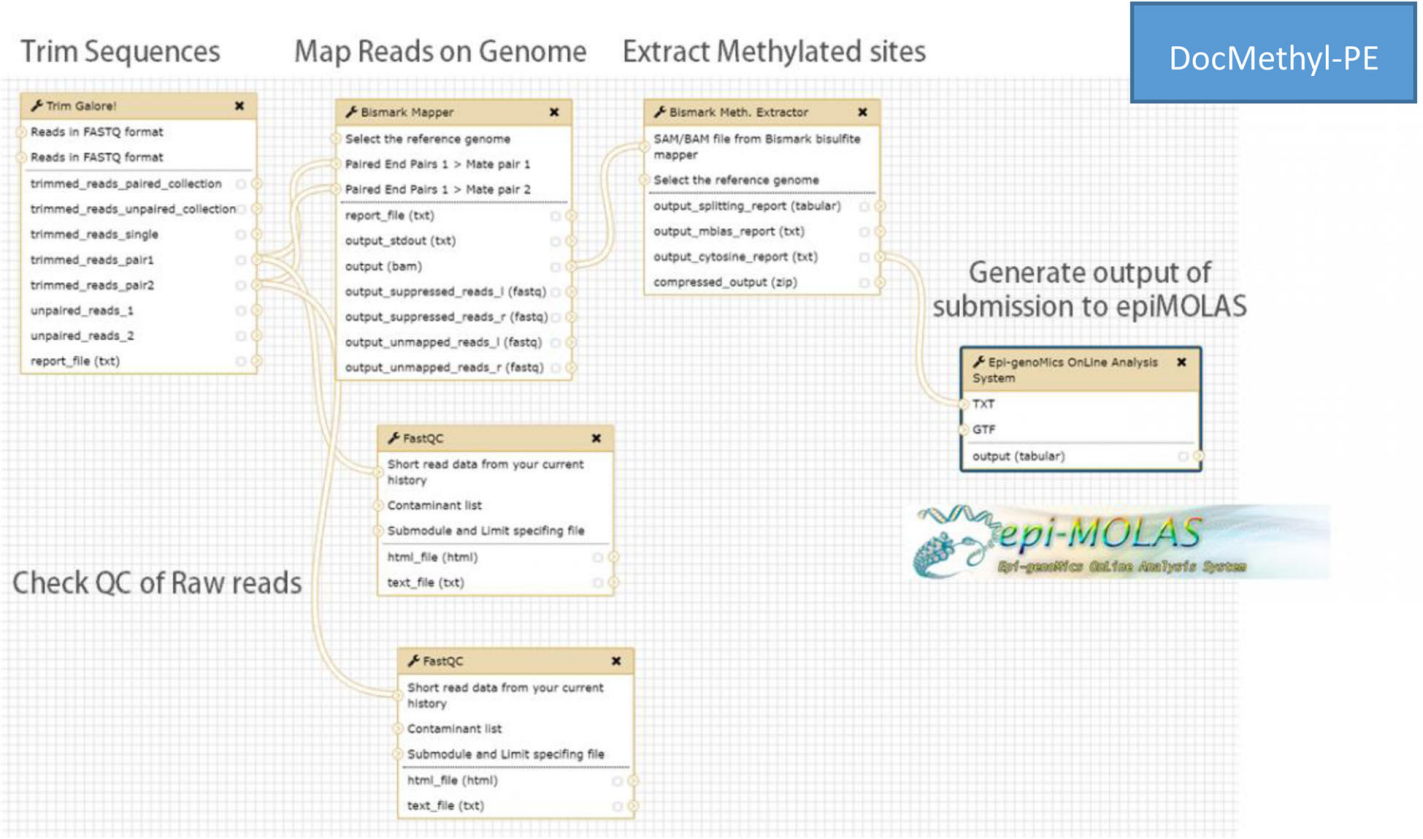
Workflow of DocMethyl-PE for WGBS paired-end data analysis, including Trim Galore, FastQC, Bismark tools, and the in-house program EpiMOLAS.jar

To run *DocMethyl*, users can directly pull down the image in a Docker runtime environment (Additional file 1 Sec. 1.2). Once *DocMethyl* is launched, Galaxy is automatically deployed, and the service is accessible through the browser-based interface. Users upload WGBS raw reads, the reference genome, and the gene annotation file for the corresponding inputs in the workflow *DocMethyl-SE* or *DocMethyl-PE* (Fig. 3 and Additional file 1 Sec. 1.4, 1.5). The output of the workflows is a tab-delimited text file, i.e. *mtable*, containing the scores for the gene-based cytosine methylation level regarding the sequence context (CG, CHG, or CHH) in the promoter and gene body regions. This seven-column data format is compatible with *EpiMOLAS_web*, enabling users to link the methylation level with the web server. One *mtable* file is generated via the workflow from one WGBS dataset. Accordingly, multiple *mtable* files for a multi-group experiment design with control conditions and experimental conditions can be uploaded under the guidance of the *EpiMOLAS_web* data submission process.

### EpiMOLAS_web

**Figure 4:**
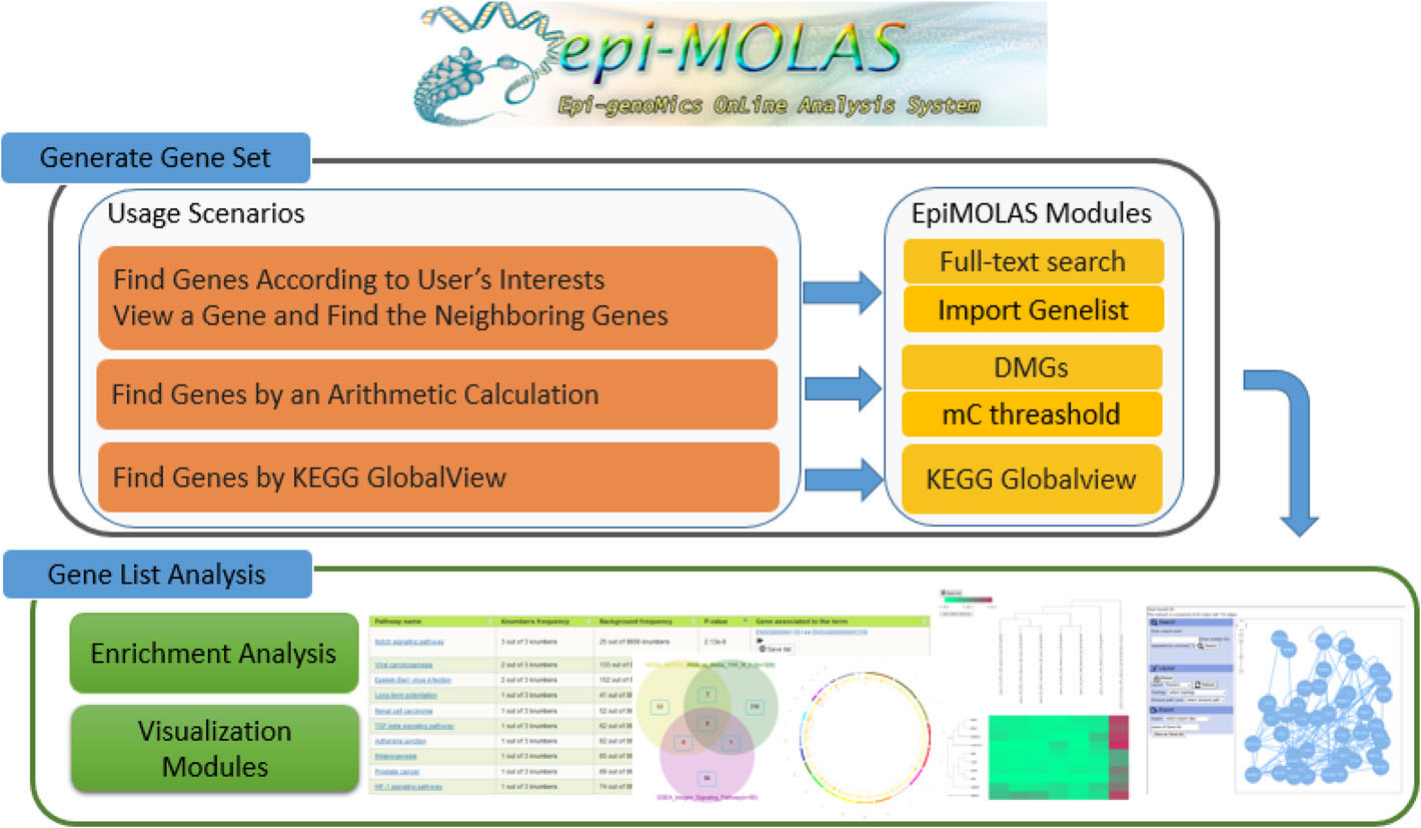
EpiMOLAS_web analysis modules with corresponding usage scenarios

*EpiMOLAS_web* provides an arithmetic calculation of the methylation level based on the experimental conditions to identify differentially methylated genes or promoters on a particular sequence context. Furthermore, several modules are available for retrieving the methylation measures of genes, such as a full-text keyword search on the Ensembl Gene ID, gene symbol, gene description, KEGG pathway name, or a batch query by Gene IDs and gene symbols (Fig. 4). The protein interaction network, hierarchical clustering heatmap, Venn diagram and Circos plot visualization modules allow users to investigate the selected gene lists in various respects. To identify the likely biological progression, the gene lists from these data retrieval approaches are used to perform GO term analysis or KEGG pathway enrichment analysis. Further details regarding *DocMethyl* and *EpiMOLAS_web* can be found in the Additional file 1, the *DocMethyl* Docker Hub repository page (https://hub.docker.com/r/lsbnb/docmethyl/), and the *EpiMOLAS_web* (http://symbiosis.iis.sinica.edu.tw/epimolas/).

### Gene set analysis and visualization

In research, the biological functions and potential mechanisms for a particular set of genes are of particular interest. Genes that associate together, therefore, may play an essential role in specific biological processes. For this reason, a gene list can be obtained from various quantitative selecting scenarios based on methylation level or from expert manually curated databases. In *EpiMOLAS_web*, we developed several modules for gene set enrichment analysis and visualization, such as KEGG pathway and GO term enrichment, histograms and boxplots of methylation levels, Venn diagrams, Circos plots, hierarchical clustering heatmaps, and protein-protein interaction networks (PPIN).

#### GO terms and KEGG pathway enrichment analysis

A set of genes of interest is usually assumed to be involved or activated in response to perturbations in specific biological processes. Through GO terms and KEGG pathway enrichment analysis, users can determine which biological processes, molecular functions, cellular components, or KEGG pathways appear to be specifically involved and have been studied in diseases, for the gene set of interest. For the enrichment score, we calculate the *p*-value based on a hypergeometric test [20].

#### PPIN analysis

We implemented a PPIN viewer to integrate, visualize, and analyze gene list members in the protein network context in the system based on Cytoscape.js, which supports network graph drawing with a force-directed layout and perturbation resilience. There are three features regarding the depiction and analysis of the interaction network: (a) search: users can search and locate the genes on the network subgraph using the gene symbols; (b) layout: the protein network layout can be displayed in Grid, Random, CoSE, Concentric, Breadthfirst, Arbor, Cola, Dagre, and Spread and with several extra network topology measures such as degree centrality, degree centrality normalized, closeness centrality, closeness centrality normalized, and betweenness centrality for network representation; (c) export: the selected gene list or network can be exported into a Cytoscape JSON file, a text file of binary protein interactions, or an image in PNG and JPG format.

#### Hierarchical clustering heatmap

The hierarchical clustering heatmap is a common unsupervised approach to show differential gene expression results. It is also a widely used visualization for displaying a table of numbers representing gene expression or methylation level. We integrated an interactive clustered heatmap visualization tool, Clustergrammer, into the system to show clusters in the methylation level of genes among samples.

#### Venn diagram and Circos plot

A Venn diagram is a simple but effective and intuitive way to examine the overlap between lists of genes. This visualization module computes the intersection of up-to four gene sets and allows users to store the results. We also integrated the Circos plot visualization module to show the location (the chromosomal coordination) of the genes in the selected list(s). This circular genome data visualization provides a different perspective of the spatial characteristics in DNA methylation across genomic regions.

## Discussion

The combination of *DocMethyl* and *EpiMOLAS_web* offers an integrated solution without tedious software installation and database management. Comparisons among several well-known platforms and tools for genome-wide DNA methylation analysis, such as BAT [29], ENCODE-WGBS [30], snakePipe [31], NGI-MethylSeq [32], Mint [33], MethylPipe [34], MethylSig [35], and Methylkit [36] with EpiMOLAS are presented in Table 1. BAT, NGI-MethylSeq and EpiMOLAS use Docker containerization technology to allow fast and simple environment deployment. Apart from EpiMOLAS and Mint, the other platforms are executed in shell scripts or workflow management systems, lacking a user-friendly web interface to satisfy the needs of laboratory researchers. Mint is accessible ins both the command line and Galaxy graphical user interface; however, it requires additional efforts to install tools and to customize the environment. Tools including ENCODE-WGBS, snakePipe, and NGI-MethylSeq have been developed to support analyses of epigenetic profiling and other -omics data (RNA-seq, ChIP-seq, Hi-C, ATAC-seq, and etc.). Nevertheless, it remains challenging to handle different -omics profiles owing to the lack of integrated analysis on heterogeneous data. Other R packages for DNA methylation analysis have community supports; however, they require external data preprocessing, read mapping and methylation calling to generate base-resolution DNA methylation data.

**Table 1:** The comparisons of EpiMOLAS with other platforms and tools on the WGBS analysis

	EpiMOLAS		BAT	ENCODE-WGBS	snakePipe	NGI-MethylSeq	Mint	MethylPipe	MethylSig	Methylkit
Environment	Docker|Galaxy web server		Docker	Shell script	Bioconda Snakemake	Docker Nextflow	Galaxy	R package	R package	R package
Sequence context	CGCHG, CHH		CG	CG, CHG, CHH	CG, CHG, CHH	CG, CHG, CHH	CG	CG, CHG, CHH	CG, CHG, CHH	CG, CHG, CHH
Start with	raw reads		raw reads	raw reads	raw reads	raw reads	raw reads	methylation call file	methylation call file	methylation call file
Docker container	***DocMethyl***	+	+	–	–	+	–	NA	NA	NA
Web interface		+ (Galaxy)	–	+	–	–	+ (Galaxy)	NA	NA	NA
Adapter and base quality trimming ^a^		+	–	+	+	+	+	NA	NA	NA
QC report ^b^		+	+	+	+	+	+	NA	NA	NA
Read mapping ^c^		+	+	+	+	+	+	NA	NA	NA
Methylation sites calling ^c^		+	+	+	+	+	+	NA	NA	NA
Discriptive statistics	***EpiMOLAS_web***	+	+	–	+	+	+	+	+	+
Find DMRs ^d^		+ (simple)	+ (metilene)	–	+ (metilene)	–	+ (DSS)	+	+	+
Clustering Analysis		+ (heatmap)	+ (heatmap)	–	+ (heatmap)	–	–	–	+ (heatmap)	–
GO term enrichment		+	–	–	–	–	–	+	–	–
KEGG pathway enrichment		+	–	–	–	–	–	–	–	–
TFBS enrichment		–	–	–	–	–	–	–	+	–
Genome-wide visualization		+ (circos plot)	+ (circos plot)	–	–	–	–	–	–	–
Interacitve Quantitative Analysis		+	–	–	–	–	–	NA	NA	NA
Data browsing and retrieving UI		+	–	–	–	–	–	NA	NA	NA
Gene list with tracking logs		+	–	–	–	–	–	NA	NA	NA
Venn analysis on gene lists		+	–	–	–	–	–	–	–	–
Interplay with other high throughput data		Protein Interactome ^e^	transcriptome	–	RNA-seq, ChIP-seq, ATAC-seq, Hi-C etc.	–	hydroxyl-methylation data	RNA-seq, ChIP-seq, DNase-seq	–	–

Remarks: ^a^EpiMOLAS, ENCODE-WGBS, snakePipe, NGI-MethylSeq and Mint adopt Trim Galore/cutadapt in the workflow for adapter and base quality trimming

^b^EpiMOLAS, snakePipe, NGI-MethylSeq and Mint integrate FASTQC to report read quality. BAT includes BSeQC for BS-seq experiment quality assessment.ENCODE-WGBS collects samtools and Bismark metrics as quality reports

^c^EpiMOLAS, ENCODE-WGBS and Mint mainly use Bismark in the workflow for alignment and methylation extraction. BAT uses segemehl and in-houseBAT_calling tools. snakePipe adopts bwa-meth for alignment and MethylDackel for methylation calling. NGI-MethylSeq provides two data analysisworkflows for choices: Bismark and bwa-meth/MethylDackel

^d^EpiMOLAS uses simple method for DMGs. BAT and snakePipe include metilene for DMRs. Mint integrates DSS for DMRs

^e^EpiMOLAS includes a dedicated plugin viewer to explore the protein interaction network

EpiMOLAS is unique among the currently available WGBS analysis platforms and tools in many aspects. Most workflows and tools provide graphical results, but they are limited to specific types of analyses. In *EpiMOLAS_web*, we designed various modules for quantitative analyses as well as a keyword-based query on the gene annotations. Taking advantage of web servers, users can explore their data in various ways and save the gene lists of each analysis with tracking logs. By utilizing BioGRID protein interaction data, we can further study the association of the genes discovered in a methylome analysis and their protein interaction network in an interactome analysis.

EpiMOLAS adopts differentially methylated genes (DMGs) instead of differentially methylated regions or cytosines (DMRs or DMCs, respectively). On the basis of DMGs, this approach would flatten the impact of DMRs into broad-scale regional signals and make it a complementary view of studying aberrant DNA methylation regions with specific genomic features base by base. Moreover, we use a straightforward approach to assess the DMGs calculated based on pairwise comparison or on a preset background subtraction. From a macro perspective, we provide a “gene-centric” approach to study the genome-wide DNA methylation changes with their potential biological functions via downstream gene set analysis.

## Conclusions

To the best of our knowledge, EpiMOLAS is the first web-based framework that adopts a two-phase approach to process WGBS raw reads and provides versatile downstream analysis, annotation, and visualization, enabling users to explore their data and obtain useful information. Docker containerization technology applied in the streamlined DNA methylation profiling workflow is not only rapid for deployment but up-scalable by increasing the number of containers running in a cloud computing environment, thereby meeting the needs of various scales of experimental design. EpiMOLAS helps users deal with their WGBS data and furthermore, alleviates the burdens of conducting reproducible analyses of publicly available data.

## Availability and requirements

**Project name:** EpiMOLAS.

**Project home page:**

http://symbiosis.iis.sinica.edu.tw/epimolas/ (EpiMOLAS_web).

https://hub.docker.com/r/lsbnb/docmethyl/ (DocMethyl).

https://github.com/markchiang/EPI-MOLAS/ (EpiMolas.jar).

**Operating system(s):** Docker containers and web application are platform-independent.

**Programming language:** Java, Python and R.

**Other requirement:** Docker installation for DocMethyl use.

**License:** Galaxy source code is licensed under the Academic Free License version 3.0.

**Any restrictions to use by non-academics:** No.

## Supplementary information

**Additional file 1.** This supplementary file provides the description and usage of DocMethyl and EpiMOLAS_web, including the installation steps on how to execute the workflow in DocMethyl and the usage guides on analyzing WGBS data through the modules in EpiMOLAS_web.

## Data Availability

Not applicable.

## Abbreviations

BS-seq: Bisulfite sequencing
DMC: Differentially methylated cytosine
DMG: Differentially methylated gene
DMR: Differentially methylated region
GO: Gene ontology
KEGG: Kyoto encyclopedia of genes and genomes
MBD-seq: Methyl-CpG-binding domain (MBD) proteins with sequencing
MeDIP-seq: Methylated DNA immunoprecipitation with sequencing
PPIN: Protein-protein interaction network
RRBS: Reduced representation bisulfite sequencing
WGBS: Whole-genome bisulfite sequencing
