# Supplementary material for: EpiMOLAS: an intuitive web-based framework for genome-wide DNA methylation analysis
Source: BMC Genomics. 2020 Apr 2;21(Suppl 3):163. doi: 10.1186/s12864-019-6404-8 (PMC7114791; doi:10.1186/s12864-019-6404-8)
Supplement: Supplementary file 1 — Additional file 1. This supplementary file provides the description and usage of DocMethyl and EpiMOLAS_web, including the installation steps on how to execute the workflow in DocMethyl and the usage guides on analyzing WGBS data through the modules in EpiMOLAS_web. [file 12864_2019_6404_MOESM1_ESM.pdf]

Supplementary Document

Content

1. Galaxy on Docker container – DocMethyl 2
   - 1.1 System Requirement 2
   - 1.2 Installation Steps....2
   - 1.3 Tools included in Galaxy/DocMethyl Workflows (Fig. S2) 3
   - 1.4 Execute the Workflow by Selection of a Suitable Dataset....6
   - 1.5 How to upload files 8
   - 1.6 Transfer Outputs to EpiMOLAS_web 9
2. EpiMOLAS_web system 10
   - 2.1 Browse existing Projects 10
   - 2.2 Create a Project....11
   - 2.3 Generate Gene Sets....15
     - 2.3.1 Find Genes According to User's Interests 16
     - 2.3.2 View a Gene and Find the Neighboring Genes....17
     - 2.3.3 Find Genes by an Arithmetic Calculation 19
     - 2.3.4 Find Genes by KEGG GlobalView....20
   - 2.4 Tools for Gene List Analysis and Visualization 20
     - 2.4.1 Gene List Enrichment Analysis....21
     - 2.4.2 Visualization Modules 22
3. Reference....29

1. Galaxy on Docker container – DocMethyl

1.1 System Requirements

DocMethyl is available at https://hub.docker.com/r/lsbnb/docmethyl/. We have tested the whole process and provided a demonstration dataset for the Galaxy Docker container on an Ubuntu (16.04 64-bit) server with four-core CPU, 16 GB of RAM, and 400 GB of data storage. The elapsed time on single thread mapping mode for the demonstration dataset (raw data size 5 GB) is about 10 hours and ~50 GB of intermediate files are generated through the workflow. We recommend more data storage for large datasets.

1.2 Installation Steps

Before starting, please have the Docker engine ready and note that all the descriptions here are the command line instructions on a daemon launched session. Check the following list:

- ✓ Visit https://docs.docker.com/install/ if you are new to DOCKER. Although different versions of the Docker engine (e.g., Windows or Mac) are available, we suggest users execute DocMethyl on a Linux environment for good efficiency and stability. Besides, a virtual machine on a private or public cloud will be a good choice for the scalability of data size.
- ✓ The server IP is required to access the Galaxy server via the web after the Docker container is launched, unless the server is directly accessed (localhost). Find the IP from the computing resource provider.

Step 1. Pull down the Galaxy Docker image from Docker Hub.

```
docker pull lsbnb/docmethyl
```

Step 2. Run the Galaxy Docker container and set port numbers for network accessibility and ftp connection.

```
docker run -d -t -i -p 8080:80 -p 8021:21 -p 8022:22 -v
$('pwd')/galaxy_guest/:/root/galaxy/database/ftp/epimolas@galaxy.or
g/ lsbnb/docmethyl /bin/bash
```

Step 3. DocMethyl now is launched and the Galaxy Server will start at localhost (DOCKER), accessible through web browser at port 8080 (Figure S1). (http://docker_IP:8080).

Step 4. Login to the Galaxy using the default user account 'epimolas@galaxy.org' and password 'epimolas', and run the built-in workflows. For Galaxy administration purposes, login to the server using the account 'admin@galaxy.org' and password 'admin@galaxy'. Please note that any manipulation of the Galaxy settings will not be carried over to a restart session of the Docker container.

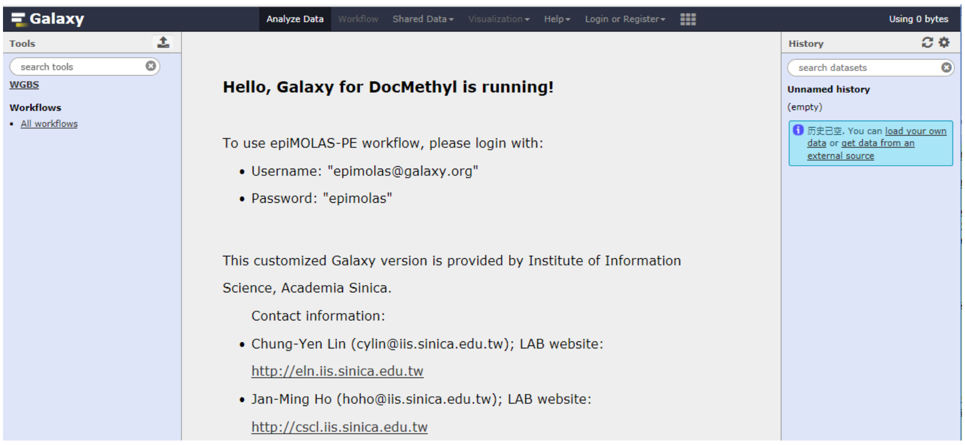

Figure S1. A snapshot on the portal page of DocMethyl Galaxy Server (http://docker_IP:8080).

1.3 Tools included in Galaxy/DocMethyl Workflows (Figure S2)

Trim Sequences: Trim Galore

(https://www.bioinformatics.babraham.ac.uk/projects/trim_galore/) – A read pair-trimming tool. It removes adapter contamination and low quality bases. Trimmed pairs containing reads less than 20 bp in with length will also be excluded.

Quality Control (QC) of Raw Reads: FastQC

(https://www.bioinformatics.babraham.ac.uk/projects/fastqc/) – It provides a trimmed read quality report that users can access quickly on screen for the read data, such as GC content, length distribution, and overrepresented sequences.

Map Reads on Genome and **Extract Methylated Sites**: Bismark

(https://www.bioinformatics.babraham.ac.uk/projects/bismark/) – A widely used bisulfite sequencing (BS-Seq) aligner that maps bisulfite-treated reads to the reference genome and extracts methylation information for individual cytosines.

Generate output of submission: EpiMolas.jar (available at http://symbiosis.iis.sinica.edu.tw/epimolas/EpiMolas.jar) – It calculates the methylation landscape on the promoter and gene body region of three sequence contexts (CG, CHG, and CHH). The methylation level is the average of the C methylation ratio from at least five observations (e.g., cytosine) on a particular region (promoter or gene body) with each observation concluded from at least four occurrences (mapping reads) (Equation 1.1 & 1.2) and writes the output, the mtable, for the web service *EpiMOLAS_web*.

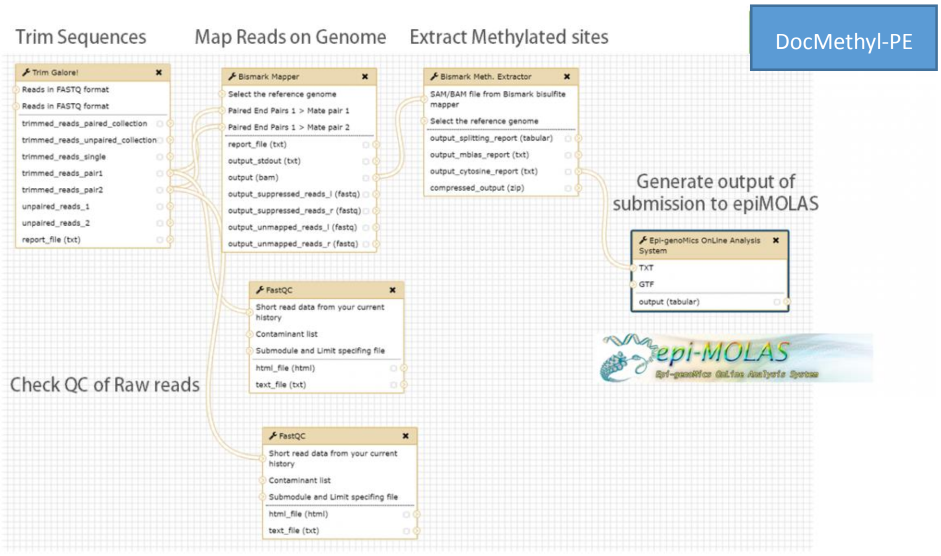

Figure S2. The whole workflow of DocMethyl-PE for paired reads.

About the mtable

5-methylcytosine (5mC) is the best known modified nucleotides in the genome. To catch the essence of the genome methylation status and to meet the efficiency for performing the analysis online, we introduced a straightforward method to measure the methylation landscapes of genes and promoters with regard to the sequence contexts.

The DNA methylation level for an individual cytosine is estimated using Equation (1.1).

$$\text{the DNA methylation level for individual cytosine } i = Ci = \frac{\#\, read\, C}{\#\, readC + readT} \qquad (1.1)$$

The methylation landscape of a promoter or a gene body is scored by the average of each observed C in all sequence contexts, as calculated using Equation (1.2):

$$\text{Average DNA methylation level in promoter or gene body} = \frac{\sum_{i \in X} c_i}{\sum_{i \in X} 1} \qquad (1.2)$$

X = promoter or gene body

For each qualified observed cytosine, its mapped read depth should reach the minimum threshold of 4 (# of read C+T for Equation 1.1). For promoter region or gene body of each gene, it should have at least five qualified observed cytosines of each sequence context type (Equation 1.2). Thus, the BS-Seq mapping report from the previous step is converted to a summary, **mtable**, of gene methylation landscapes scored by six measurements (*i.e.,* pmt_CG, pmt_CHG, pmt_CHH, gene_CG, gene_CHG, gene_CHH) (Figure S3 & S4). The threshold of read depth, upstream and downstream of promoter regions can be adjusted according to sequencing depth of coverage and putative functional region upstream from the gene.

1	2	3	4	5	6	7
gene_id	pmt_CG	gene_CG	pmt_CHG	gene_CHG	pmt_CHH	gene_CHH
ENSG00000254870	0.000000	0.276349	0.000000	0.007246	0.046667	0.056741
ENSG00000198563	NaN	0.258258	0.000000	0.008230	0.002304	0.063635
ENSG00000234006	0.000000	NaN	0.000000	NaN	0.083333	NaN
ENSG00000213760	NaN	0.000000	NaN	0.000000	NaN	0.046667
ENSG00000204498	NaN	NaN	0.000000	0.000000	0.000000	0.031111
ENSG00000226979	NaN	NaN	0.055556	0.000000	0.050725	0.000000
ENSG00000232810	NaN	0.482143	0.036706	0.010417	0.171364	0.086735
ENSG00000227507	NaN	0.285714	0.125000	0.000000	0.132344	0.035088
ENSG00000204482	NaN	0.937500	NaN	0.005882	NaN	0.035122
ENSG00000204475	NaN	NaN	NaN	0.000000	NaN	0.062500
ENSG00000230622	NaN	NaN	0.164454	0.000000	0.338504	0.000000

Figure S3. An example of mtable

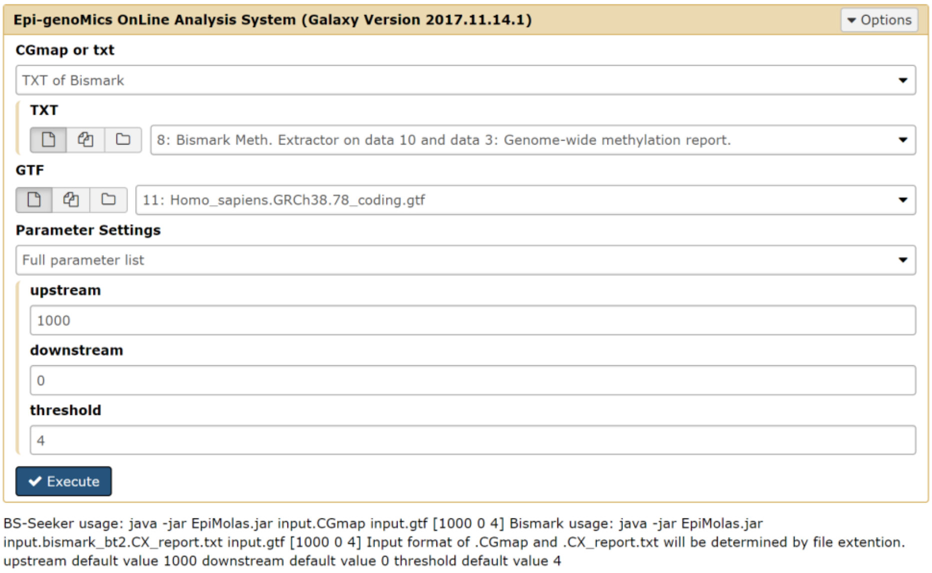

Figure S4. Parameter setting window of EpiMolas.jar in DocMethyl. The default promoter definition is the upstream 1000 bases to 0 away from the transcription start site, and calculation of the methylation ratio is performed only for Cs with a mapping depth greater than 4. Three sequence contexts (CG, CHG, and CHH) in the promoter and gene body regions are reported when at least five Cs are scored.

1.4 Execute the Workflow by Selection of a Suitable Dataset

We built two Galaxy workflows to meet the need to process raw data in paired-end format (DocMethyl-PE) or single end (DocMethyl-SE) format respectively. These two workflows can be found in "WGBS" of the menu (left panel) in Galaxy/ DocMethyl. To run the workflow, users should specify read files and the target genome information (the genome sequences in fasta and genome's annotation in gtf) for the run (Figure S5) in the dialog box and submit the job to Galaxy server. Please make sure that the raw files are in a normal FASTQ format. Compressed read files in format *.gz or *.bz2 are acceptable. Once a DocMethyl job starts, the steps of the query will be listed in right panel on the Galaxy web interface (Figure S6).

A galaxy workflow can take files from client desktop; however, this is not applicable in most cases. To use big files in Galaxy and DocMethyl, please refer to the next Section (1.5 How to upload files) for guidance.

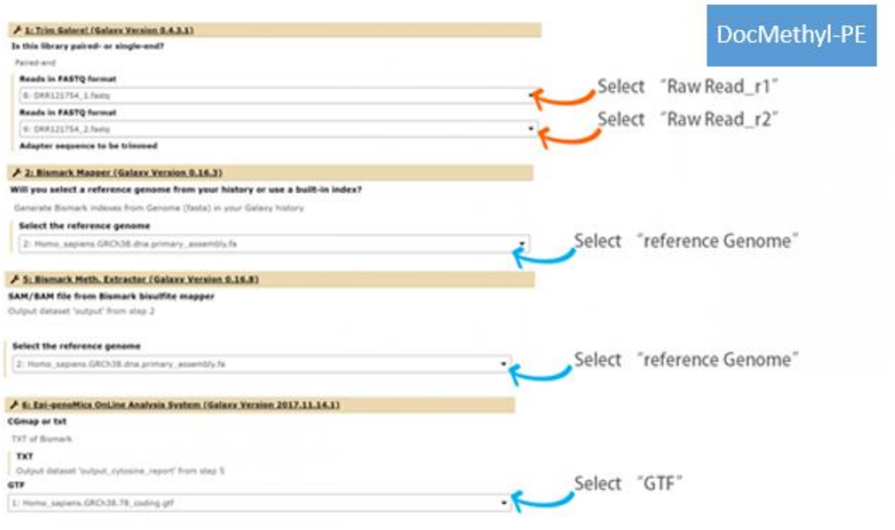

Figure S5. Selecting the files required for a DocMethyl-PE run.

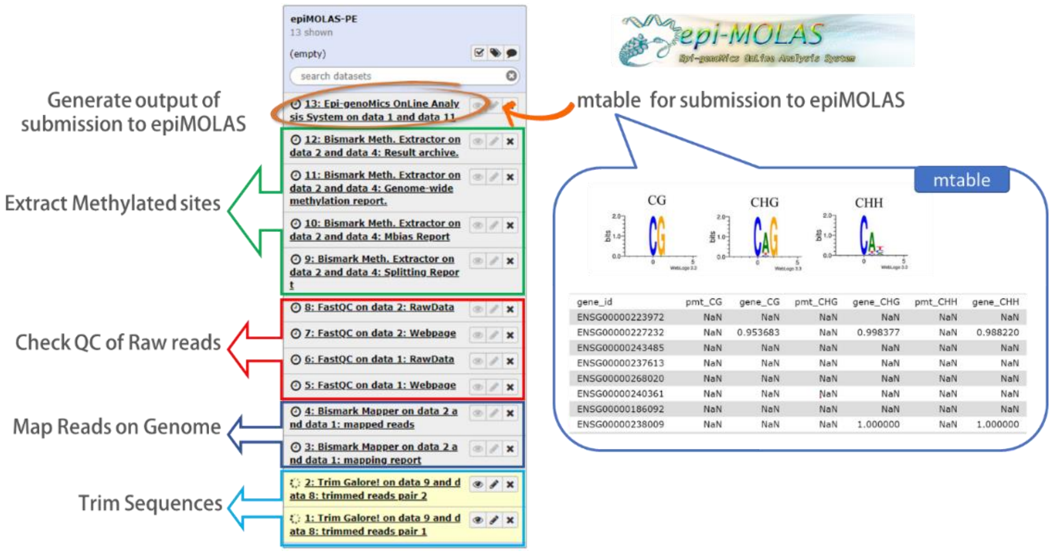

Figure S6. Scheduled processes of a typical run in the Galaxy workflow. Galaxy controls all the processes of a submitted job from trimming the input raw reads to the end output *.mtable file. The progress of the job, one step after another, is shown with color coding for scheduled (grey), in progress (light yellow), and completed (light green). The content of the output mtable (step 13, Epi-genoMics OnLine Analysis System on data 1 and data 11 in this case), generated via the DocMethyl paired-end (DocMethyl-PE) workflow, includes six measurements of C methylation regarding the location (promoter region and gene body) and sequence types (CG, CHG, CHH).

1.5 How to upload files

There are complicated and diverse settings to make the Docker-wrapped Galaxy default FTP server work in various network environments. Therefore, we provided two shortcut solutions to use large files in Galaxy/ DocMethyl without manipulating the system configurations. Considering that most WGBS analyses require a deep read coverage of the genome, which is above the limit of file size in a browser uploading session (around 2 GB), we suggest users can go straight to Solution B, especially B2, to use files uploaded in the same server that hosts the DocMethyl Docker.

Solution A. For files with a size less than 2 GB, find "WGBS" in the Galaxy menu panel and use "upload file/ choose local file" to send the file to the server.

Solution B. For files > 2 GB (most cases), you can choose one of the two approaches below:

B1. For data deposited in **an open-access ftp site**, find "WGBS" in the Galaxy menu panel, choose "upload file -> paste and fetch data" and provide the file URL(s) to get the file(s).
Example: ftp://ftp.ddbj.nig.ac.jp/ddbj_database/dra/fastq/SRA068/SRA068307/SRX247357/SRR776587_1.fastq.bz2

B2. Connect to the Linux server that hosts the DocMethyl Docker container via a FTP software tool. Create a file directory "galaxy_guest" in the same folder the Docker image being deployed. For example, if "docker pull" command is executed in the path "$~/home/user/test_docker/", the correct path of the FTP folder will be "$~/home/user/test_docker/galaxy_user/". This folder will be mounted as the deployed DocMethyl/ Galaxy FTP default file folder. Now, from the "upload file -> choose FTP file" in "WGBS" on the Galaxy menu (the left panel of the browser interface), you can find the files that were uploaded into this /galaxy_user/ folder (Figure S7).

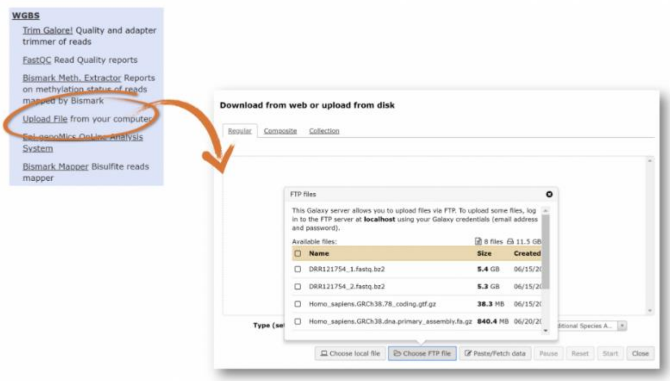

Figure S7. Find files in a local directory that is mounted as a FTP directory.

1.6 Transfer Outputs to EpiMOLAS_web

As shown in Figure S6, a DocMethyl job triggers thirteen continuous steps and produces the output mtable in the last step. One mtable file will be derived from one submitted BS-Seq/ WGBS read dataset. For example, if an experimental design includes two conditions with three replicates each, there will be six mtables in total derived from six DocMethyl jobs of six WGBS datasets. Users can download the mtable of each job from Galaxy right panel; these files are compatible with the EpiMOLAS web application, EpiMOLAS_web. For users who run the DocMethyl workflow on a genome other than human, mouse, or Arabidopsis, the bisulfite mapping reports from Bismark are available in the previous step (*i.e.*, step 12 in Figure S6). Please note that these files may be large and will take a longer time to download.

2. EpiMOLAS_web system

EpiMOLAS_web (http://symbiosis.iis.sinica.edu.tw/epimolas) is a web service that links the summary of WGBS data (*mtable*) with a rich annotation databases for human, mouse, and Arabidopsis (Figure S8). The data uploading process is a wizard guided method that leads users to create a private or open-accessible website in EpiMOLAS_web. One important issue is the compatibility of user's data. The format *mtable* is described in Section 1.3. For users who does not use the Docker container DocMethyl (https://hub.docker.com/r/lsbnb/docmethyl/), please check the usage of "*EpiMolas.jar*" to generate a mtable through BS-Seq mapper BS-Seeker2/bs_seeker2-call_methylation.py or Bismark/bismark_methylation_extractor programs (http://symbiosis.iis.sinica.edu.tw/epimolas/mapping.html), and make sure that the reference genome and gtf file are compatible with EpiMOLAS_web (http://symbiosis.iis.sinica.edu.tw/epimolas/gtf.html). A web project built for non-registered users is held in the system for one month. Long term website maintenance is also available upon request.

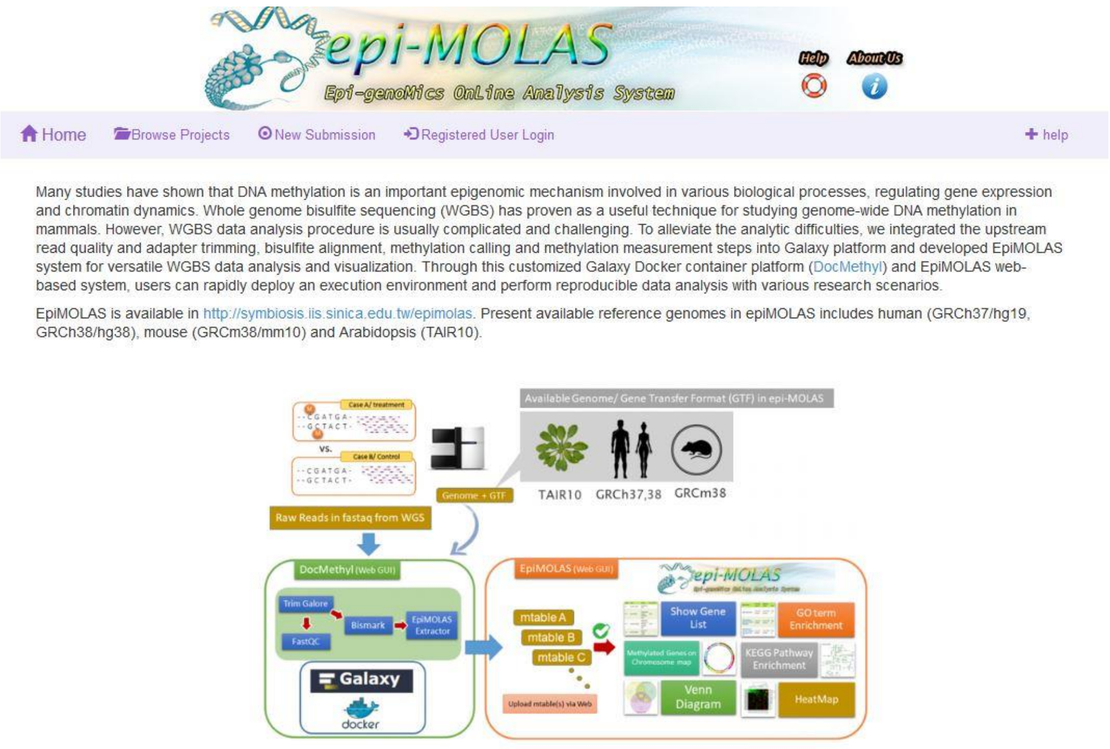

Figure S8. The web portal of EpiMOLAS_web.

2.1 Browse existing Projects

Before users' data becomes ready, there are Demo Sites (human, mouse,

and Arabidopsis) that allow users to try the analysis, or users can find other established projects in the system if the project owners (data submitters) set the website release status as "open to public" (Figure S9 & S10).

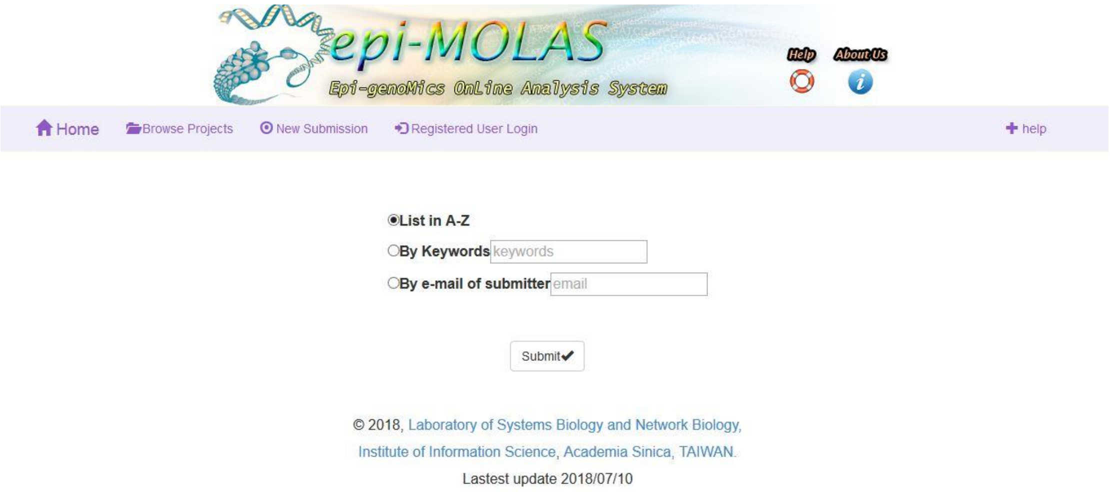

Figure S9. The web portal for browsing projects.

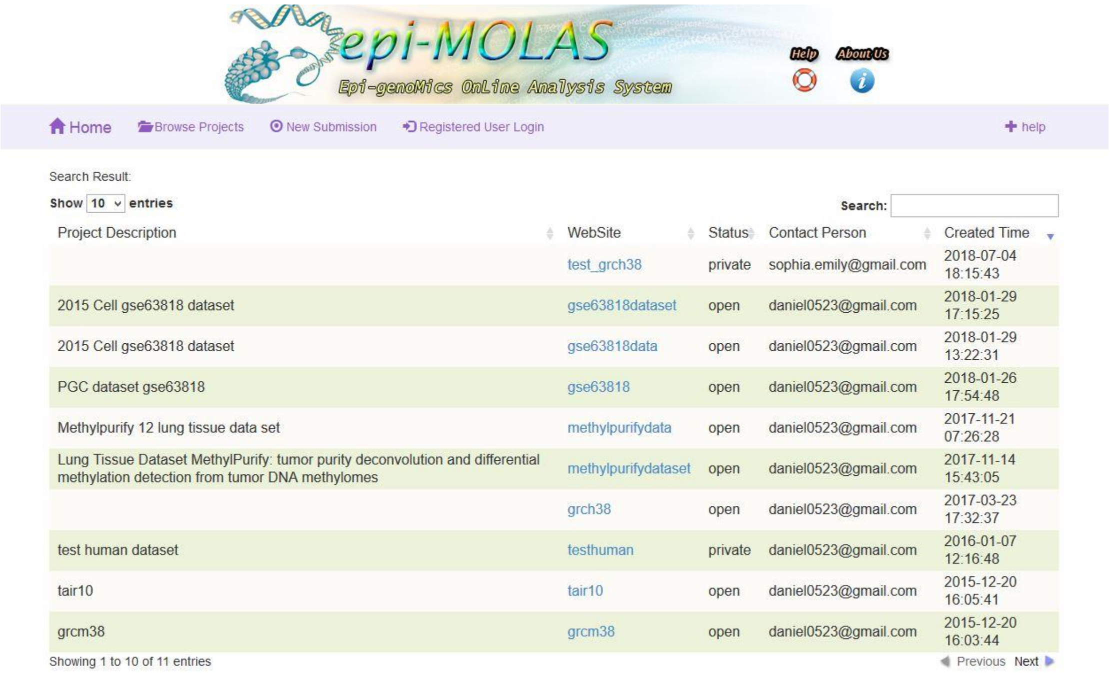

Project Description	WebSite	Status	Contact Person	Created Time
	test_grch38	private	sophia.emily@gmail.com	2018-07-04 18:15:43
2015 Cell gse63818 dataset	gse63818dataset	open	daniel0523@gmail.com	2018-01-29 17:15:25
2015 Cell gse63818 dataset	gse63818data	open	daniel0523@gmail.com	2018-01-29 13:22:31
PGC dataset gse63818	gse63818	open	daniel0523@gmail.com	2018-01-26 17:54:48
Methylpurify 12 lung tissue data set	methylpurifydata	open	daniel0523@gmail.com	2017-11-21 07:26:28
Lung Tissue Dataset MethylPurify: tumor purity deconvolution and differential methylation detection from tumor DNA methylomes	methylpurifydataset	open	daniel0523@gmail.com	2017-11-14 15:43:05
	grch38	open	daniel0523@gmail.com	2017-03-23 17:32:37
test human dataset	testhuman	private	daniel0523@gmail.com	2016-01-07 12:16:48
tair10	tair10	open	daniel0523@gmail.com	2015-12-20 16:05:41
grcm38	grcm38	open	daniel0523@gmail.com	2015-12-20 16:03:44

Figure S10. List of all projects stored in EpiMOLAS_web with their status and contact information.

2.2 Create a Project

Mtables generated by the DocMethyl workflow in the Galaxy/DocMethyl Docker or by EpiMolas.jar alone can be submitted to the EpiMOLAS_web server to create a project

which is a website that joins the data to the annotation database and the analysis toolkit. The six measurements are summarized by the combination of three sequence contexts (CG, CHG, and CHH) and two gene regions (promoter and gene body) (Figure S6). The general principle is depicted in Figure S11.

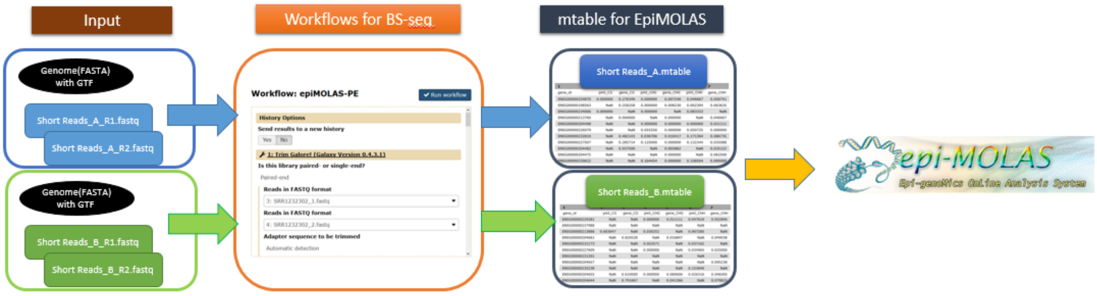

Figure S11. Illustration of throughput analysis from raw data to creating a project.

The option "**New Submission**" can be found on the navigator bar. Users should choose the compatible genome and load files first. Clicking on the "demo" icon beside the genome items will start a short tutorial about the data uploading process (Figure S12). Alternatively, users can try the "Load example data" button beside the dataset uploading box and use the demo dataset to run through the data deployment process to the resulting website. Gene IDs in each uploaded mtable will be matched to the EpiMOLAS_web database and return the matching rate upon the file uploading process; the sample label should be assigned here. Click on “submit” and the server will generate a brief summary of this submission (Figure S13). For a registered user, we require the access authority in the next step, then users will be lead to subsequent steps to fill in all the required information, *e.g.*, registered user’s email, a password (for validating this registered user), the website access policy (*i.e.*, open to public, private use, or restricted to people with a secret word shared by the project or website creator). For non-registered users, less information is required, however, there is also no detailed website policy options available; therefore, the created website will be held for one month only. When all the required information has been provided and submitted, system will start processing the data linkage and construct a web portal for this submission (Figure S14 & S15).

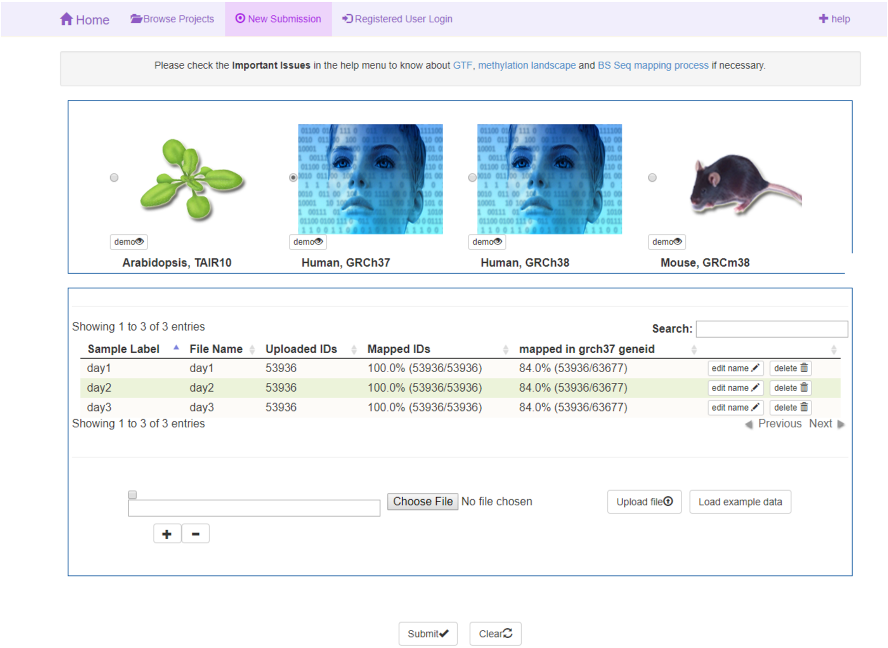

Figure S12. Creating a project.

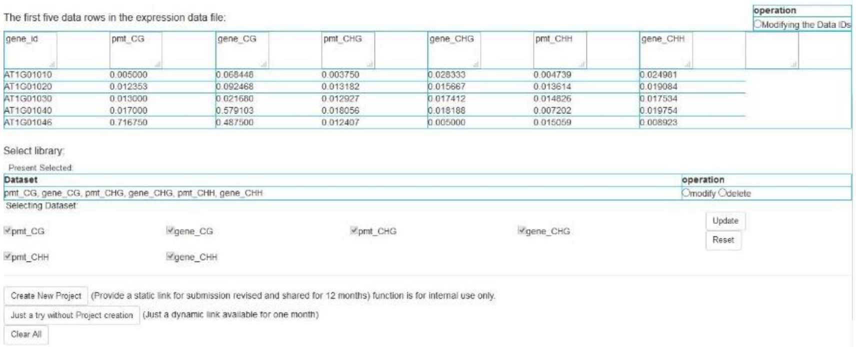

Figure S13. Summary of the uploaded mtables in the submission.

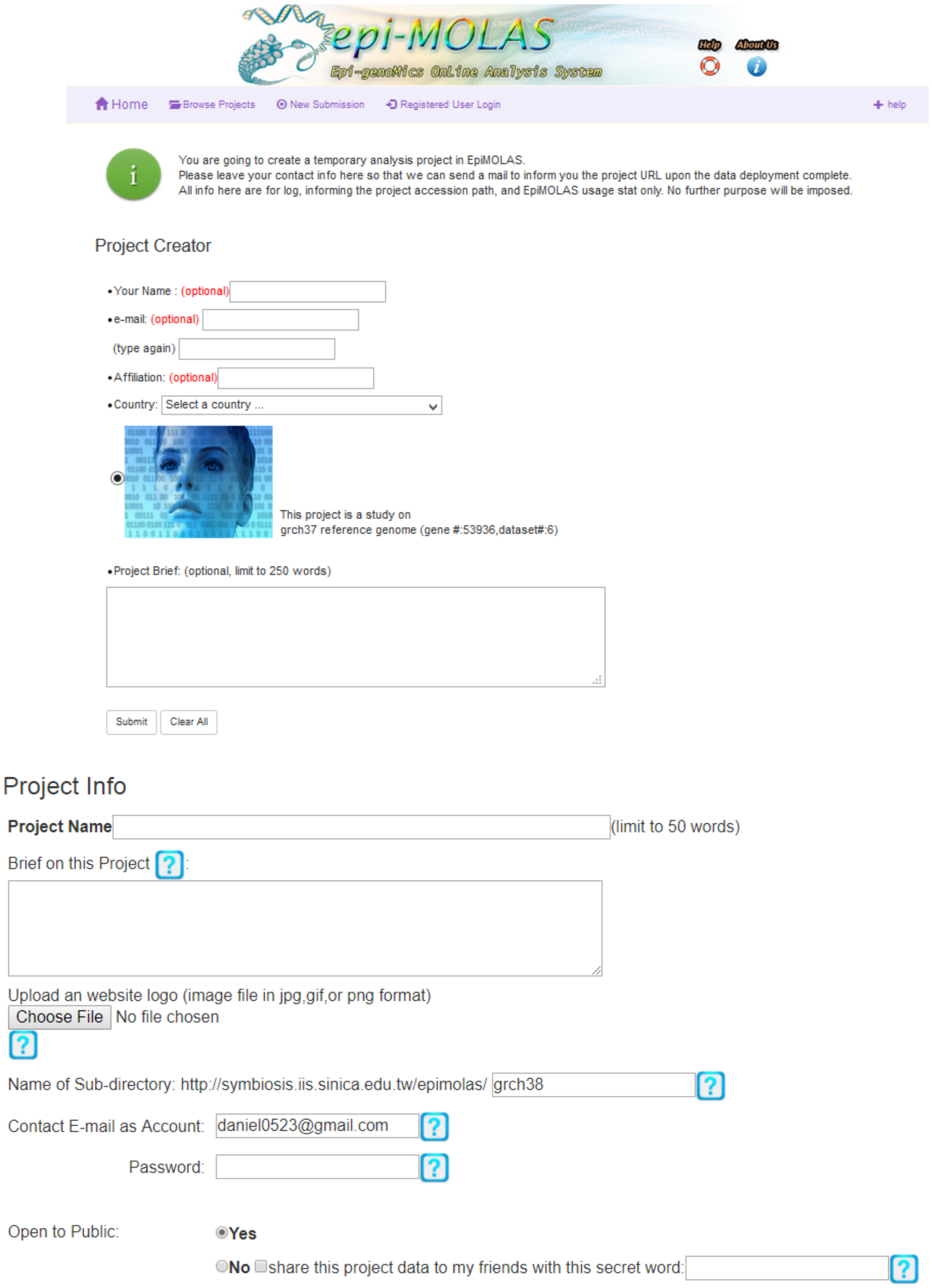

Figure S14. The web portal allows users to provide information about the project and set the project as public or private.

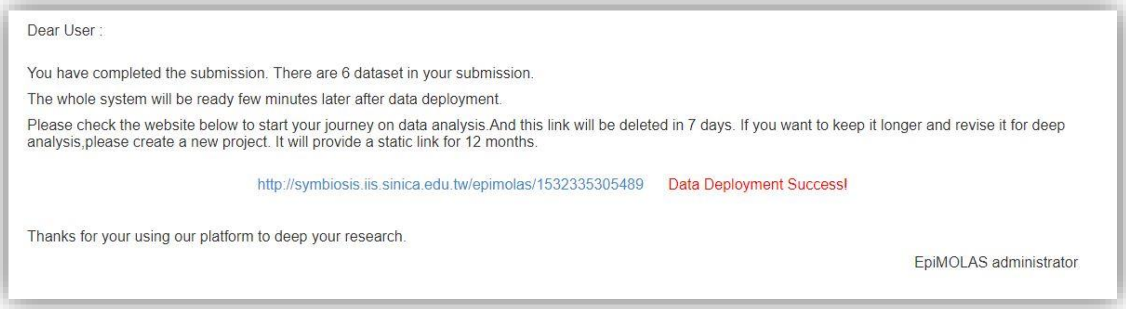

Dear User :

You have completed the submission. There are 6 dataset in your submission.

The whole system will be ready few minutes later after data deployment.

Please check the website below to start your journey on data analysis.And this link will be deleted in 7 days. If you want to keep it longer and revise it for deep analysis,please create a new project. It will provide a static link for 12 months.

http://symbiosis.iis.sinica.edu.tw/epimolas/1532335305489 Data Deployment Success!

Thanks for your using our platform to deep your research.

EpiMOLAS administrator

Figure S15. Message from the administrator to notify the completion of a job submission.

For more details about new project creation, please visit our online documentation. http://symbiosis.iis.sinica.edu.tw/epimolas/build_project.html

Once the project is created successfully, please check the online tutorials on how to access and investigate the data.
http://symbiosis.iis.sinica.edu.tw/epimolas/access_project.html

2.3 Generate Gene Sets

A general strategy to explore biological meaning in high-throughput, genome-wide scale experiments is to find a set of genes that is associated with a particular function. The gene set may be derived from a quantitative assay based on a comparison, for example, to find genes that meet the criteria of "having difference between two experiment groups", such as a cut-off ratio or a subtraction result. Other ways to define gene sets are canonically defined sets of genes such as gene ontology (GO) terms, Kyoto Encyclopedia of Genes and Genomes (KEGG) pathways, and Boolean operations on an interesting collection from users' domain knowledge. In this case, the gene lists are often cross-referred to quantitative assay-derived gene sets to highlight the genes with dynamics among the experimental design. Here we design various ways to meet these needs. Besides the arithmetic calculation on values (the six methylation measures), users can extract gene sets according to their interests, such as a text or keyword search on annotation tables, or a KEGG GlobalView query, or finding genes by uploading a list. (Figure S16).

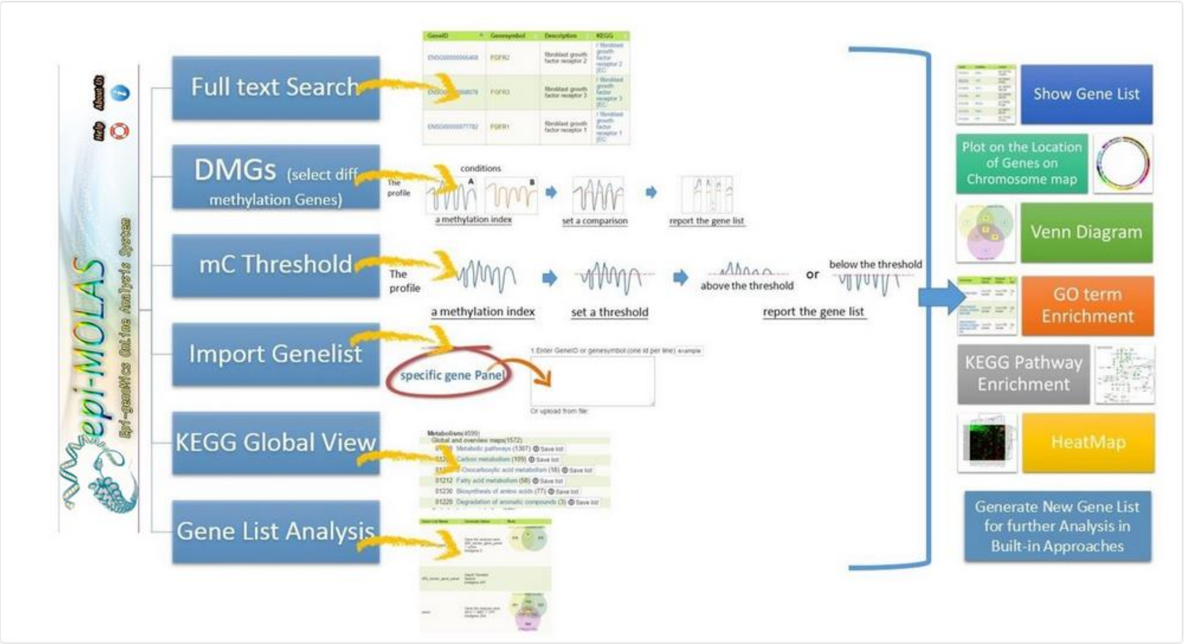

Figure S16. The schema of the EpiMOLAS_web analysis modules. It provides ways for users to decipher their data more intuitively and generate a gene list for specific spatiotemporal scenarios. These methods are described in this supplementary document and at the website in more detail.

2.3.1 Find Genes According to User's Interests

In the module "Full-Text Search", users can search the annotation database by Gene ID, gene symbol, keywords in the gene description, and by KEGG pathway name. In the modules "KEGG GlobalView", users can browse KEGG pathway lists and find genes on the interactive map. The function "save as a list" in the pathway list view is available to grasp the genes in a KEGG map as a gene list for other analyses (Figure S17).

In the module "Import Genelist", users can copy and paste a list into the query form or can upload a plain text file containing a gene list in Ensembl gene IDs or official gene symbols.

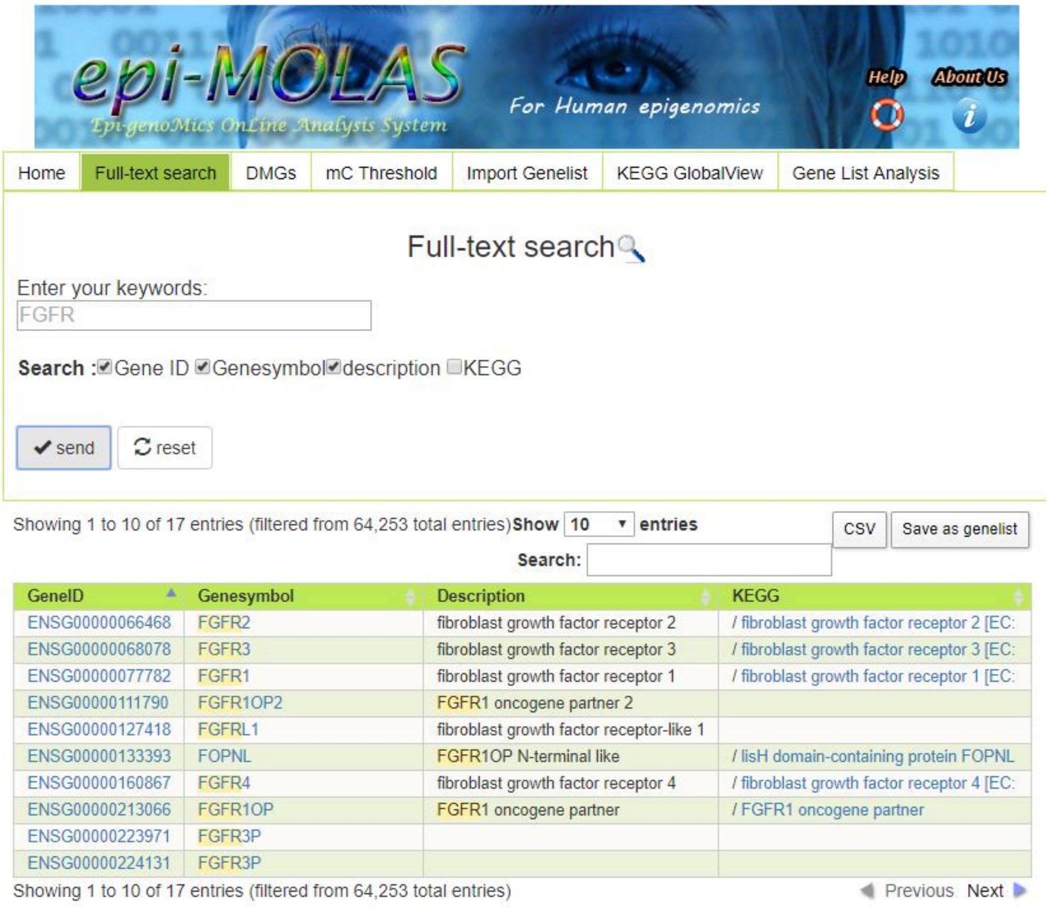

Figure S17. An example of a full text search on the annotation database.

2.3.2 View a Gene and Find the Neighboring Genes

Figure S18 shows the gene information provided in EpiMOLAS_web, including the basic description in the database, and the six methylation measures of this gene among all the experiment conditions or replicates. A Genome browser view for the location and gene structure, and an interactive chart to list the gene's neighbors in a given range are also provided.

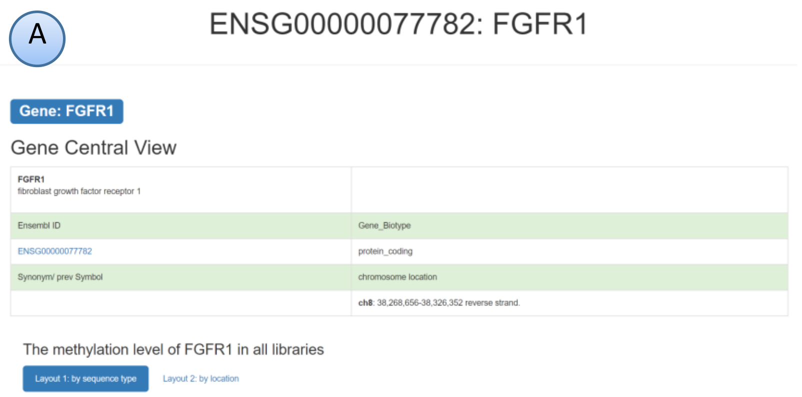

B

Layout 1: by sequence type

Layout 2: by location

Layout 1

Main categories in methylC sequence contexts (CG/CHG/CHH)

Methylation Level						
ENSG00000077782	pmt_CG	gene_CG	pmt_CHG	gene_CHG	pmt_CHH	gene_CHH
PGC_10W_embryo1_M_HiSeq2000	NA	0.070668	NA	0.020021	0.025	0.016359
PGC_11W_embryo1_F_HiSeq2500	NA	0.081937	0.042857	0.016529	0.032483	0.01041
PGC_11W_embryo1_M_rep1_HiSeq2000	NA	0.040711	NA	0.028143	NA	0.017666
PGC_11W_embryo1_M_rep2_HiSeq2000	NA	0.069613	NA	0.022122	NA	0.012733
PGC_17W_embryo1_F_HiSeq2500	NA	0.057607	0.046429	0.018839	0.008471	0.011115
PGC_19W_embryo2_M_HiSeq2500	NA	0.006452	NA	0.017344	NA	0.008884
Soma_11W_embryo1_M_HiSeq2500_deeper	NA	0.768997	NA	0.029733	NA	0.031864

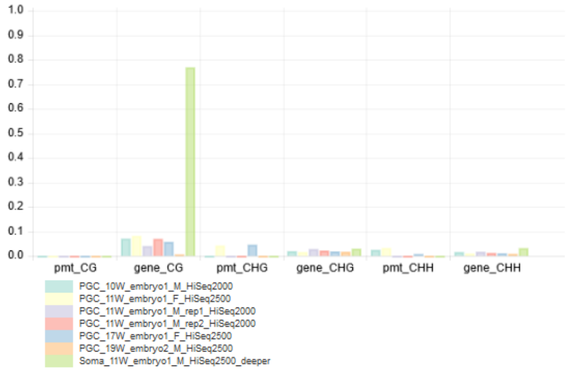

Layout 1: by sequence type

Layout 2: by location

Layout 2

Main categories in methylC location (promoter / gene)

Methylation Level						
ENSG00000077782	pmt_CG	pmt_CHG	pmt_CHH	gene_CG	gene_CHG	gene_CHH
PGC_10W_embryo1_M_HiSeq2000	NA	NA	0.025	0.070668	0.020021	0.016359
PGC_11W_embryo1_F_HiSeq2500	NA	0.042857	0.032483	0.081937	0.016529	0.01041
PGC_11W_embryo1_M_rep1_HiSeq2000	NA	NA	NA	0.040711	0.028143	0.017666
PGC_11W_embryo1_M_rep2_HiSeq2000	NA	NA	NA	0.069613	0.022122	0.012733
PGC_17W_embryo1_F_HiSeq2500	NA	0.046429	0.008471	0.057607	0.018839	0.011115
PGC_19W_embryo2_M_HiSeq2500	NA	NA	NA	0.006452	0.017344	0.008884
Soma_11W_embryo1_M_HiSeq2500_deeper	NA	NA	NA	0.768997	0.029733	0.031864

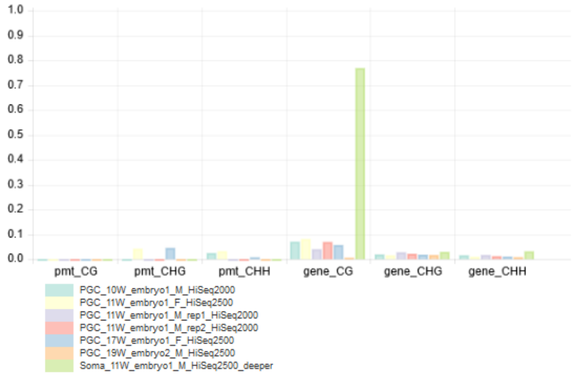

C

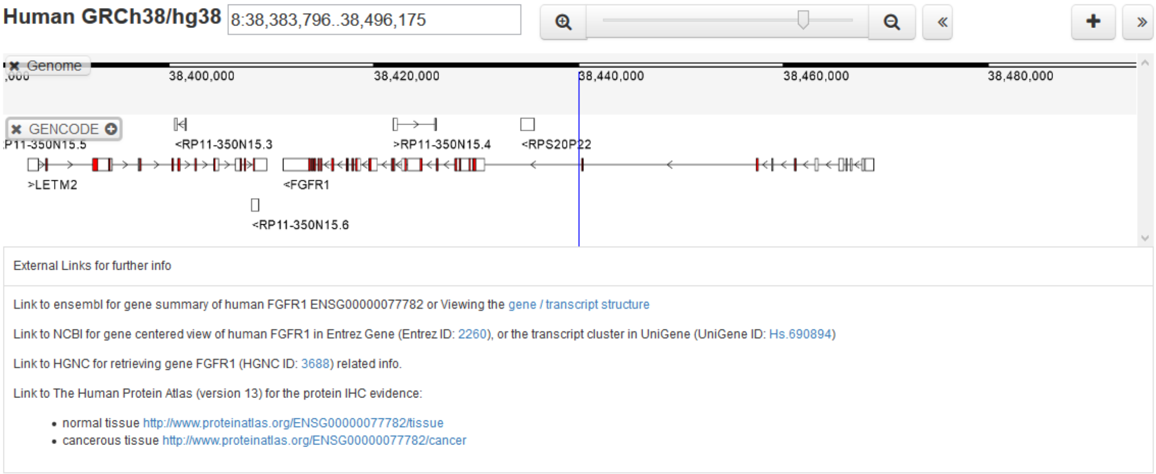

D

Neighboring Genes

Drop down the list to select a range. Given this gene, its downstream (e.g. - 1Kb) and upstream (e.g. + 1Kb) flanking genes are extracted as a list.

100 Kb ▼

Neighboring Genes (-100000 bps ~ +100000 bps)				Save list
1	ENSG00000147548 | [Ensembl]	WHSC1L1	protein_coding	8: 38127215 - 38239790 , reverse strand
2	ENSG00000254898 | [Ensembl]	RP11-513D5.2	antisense	8: 38193499 - 38195069 , forward strand
3	ENSG00000272092 | [Ensembl]	RP11-350N15.5	lincRNA	8: 38239882 - 38240979 , forward strand
4	ENSG00000165046 | [Ensembl]	LETM2	protein_coding	8: 38243725 - 38267045 , forward strand
5	ENSG00000254981 | [Ensembl]	RP11-350N15.3	antisense	8: 38258054 - 38259201 , reverse strand
6	ENSG00000272159 | [Ensembl]	RP11-350N15.6	antisense	8: 38265566 - 38266260 , reverse strand
7	ENSG00000077782 | [Ensembl]	FGFR1	protein_coding	8: 38268656 - 38326352 , reverse strand
8	ENSG00000255201 | [Ensembl]	RP11-350N15.4	antisense	8: 38279407 - 38283614 , forward strand
9	ENSG00000239218 | [Ensembl]	RPS20P22	pseudogene	8: 38291865 - 38293182 , reverse strand
10	ENSG00000196166 | [Ensembl]	C8orf86	protein_coding	8: 38368352 - 38386180 , reverse strand
11	ENSG00000253361 | [Ensembl]	RP11-675F6.3	lincRNA	8: 38401170 - 38410198 , reverse strand
12	ENSG00000254100 | [Ensembl]	RP11-675F6.4	lincRNA	8: 38409766 - 38416538 , reverse strand

Figure S18. (A) The basic annotation with the chromosome location. (B) The methylation levels for the promoter and gene body with three types of methylation, CG, CHG, and CHH. (C) The genome browser to navigate the gene on the genome. (D) The neighboring genes around a specific gene (ENSG00000077782 in this case is marked in red).

2.3.3 Find Genes by an Arithmetic Calculation

Module **DMGs** (differentially methylated genes) is a pairwise comparison workflow between two data pools, where single or multiple datasets can be assigned to one data pool (e.g., an experimental condition). Through customized and flexible parameter settings, genes that fulfill the criteria are selected. Module **mC Threshold** is used to select genes above or below a cutoff in at least one dataset, or among all datasets. These two modules provide different ways to extract the methylation signatures of the six combinatorial sequence contexts and regions.

2.3.4 Find Genes by KEGG GlobalView

KEGG Pathway maps provide users with a knowledge-based view of biological processes. Users can find a KEGG map by a pathway name search or by browsing. In a KEGG pathway map, each KEGG component box highlighted in green means that the content of this KEGG component matches the user's uploading data. Clicking on the component will lead to a summary page of the methylation landscapes of the genes (gene body and promoter for CG, CHG, CHH) for all mapped genes in this component (Figure S19). In addition, users can store gene sets in specific KEGG maps and perform versatile gene set analyses as described previously (See **2.3.1 Find Genes According to User's Interests**).

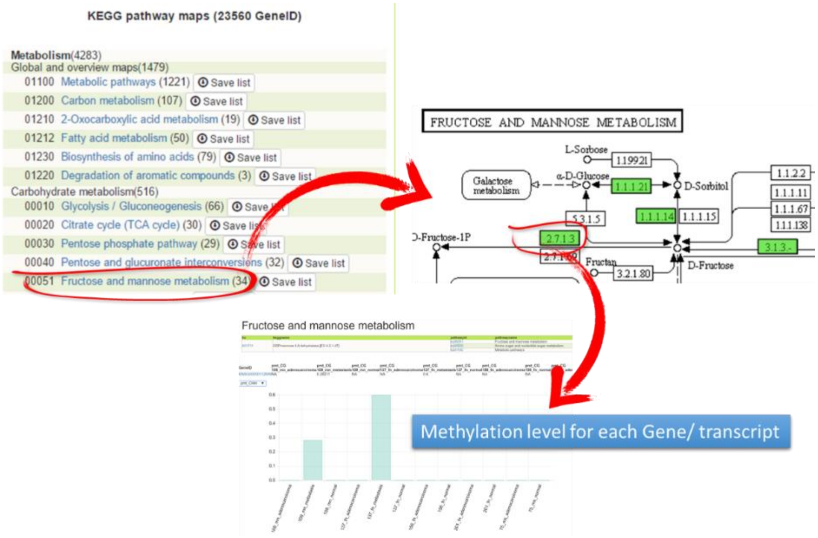

Figure S19. Finding genes by a specific pathway view. A partial list shown in the module "**KEGG GlobalView**". Click on the pathway link, such as "00051 Fructose and mannose metabolism" in this case, and system will return the corresponding KEGG map. Users can examine the methylation landscapes of genes for each map component.

2.4 Tools for Gene list Analysis and Visualization

Gene list items are derived from all kinds of sources, such as an arithmetic result on a particular feature between two groups (*i.e.*, calculated in the module "DMG" interface), or a keyword present in the annotation table (*i.e.*, full-text search). A gene list that provides clear biological insight will be good evidence for the hypothesis of the original experimental design. In EpiMOLAS_web, we built gene set tools for enrichment analysis and visualization, such as KEGG pathway enrichment, GO term enrichment, circos plot, heatmap with hierarchical clustering, and protein-protein interaction

networks, for a macro-level view. A gene list from any data analysis module can be displayed and saved for later reuse. One analysis function, *Venn diagram* is only available for the module “**Gene List Analysis”** (Figure S20).

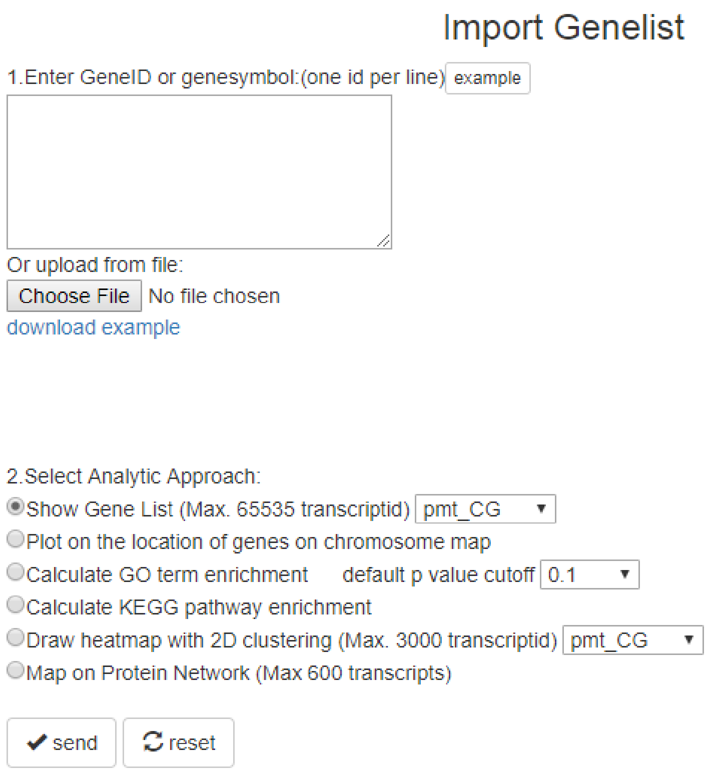

Figure S20. The gene set analysis modules. Several approaches, such as GO terms and KEGG pat hway enrichment analysis, hierarchical clustering heatmap, and protein interaction network analysis, are provided for gene list analysis.

2.4.1 Gene List Enrichment Analysis

Gene Ontology terms and KEGG pathway enrichment analysis

In general, a set of genes of interest is usually involved in some activities for responding to perturbations of particular biological processes. Users can study which biological processes, molecular functions, cellular components, or KEGG pathways are associated with a particular disease or a specific phenotype through GO terms and KEGG pathway enrichment analysis. For the statistical significance of enrichment score, we adopt the hypergeometric probability distribution to calculate the p-value. Figure S21 and S22 are examples for a given gene list run for GO and KEGG enrichment. The “save list” in the right end column will extract a sub-list of genes that were submitted for GO enrichment and are associated with the GO functional categories.

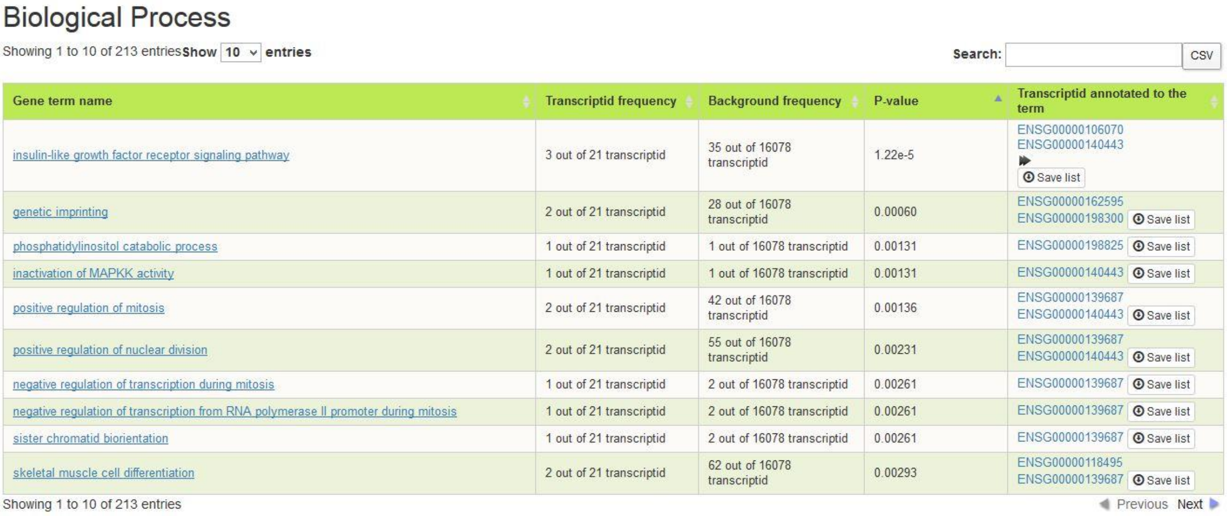

Biological Process

Showing 1 to 10 of 213 entries Show 10 entries

Search: CSV

Gene term name	Transcriptid frequency	Background frequency	P-value	Transcriptid annotated to the term
insulin-like growth factor receptor signaling pathway	3 out of 21 transcriptid	35 out of 16078 transcriptid	1.22e-5	ENSG00000106070 ENSG00000140443 Save list
genetic imprinting	2 out of 21 transcriptid	28 out of 16078 transcriptid	0.00060	ENSG00000162595 ENSG00000198300 Save list
phosphatidylinositol catabolic process	1 out of 21 transcriptid	1 out of 16078 transcriptid	0.00131	ENSG00000198825 Save list
inactivation of MAPKK activity	1 out of 21 transcriptid	1 out of 16078 transcriptid	0.00131	ENSG00000140443 Save list
positive regulation of mitosis	2 out of 21 transcriptid	42 out of 16078 transcriptid	0.00136	ENSG00000139687 ENSG00000140443 Save list
positive regulation of nuclear division	2 out of 21 transcriptid	55 out of 16078 transcriptid	0.00231	ENSG00000139687 ENSG00000140443 Save list
negative regulation of transcription during mitosis	1 out of 21 transcriptid	2 out of 16078 transcriptid	0.00261	ENSG00000139687 Save list
negative regulation of transcription from RNA polymerase II promoter during mitosis	1 out of 21 transcriptid	2 out of 16078 transcriptid	0.00261	ENSG00000139687 Save list
sister chromatid biorientation	1 out of 21 transcriptid	2 out of 16078 transcriptid	0.00261	ENSG00000139687 Save list
skeletal muscle cell differentiation	2 out of 21 transcriptid	62 out of 16078 transcriptid	0.00293	ENSG00000118495 ENSG00000139687 Save list

Showing 1 to 10 of 213 entries Previous Next

Figure S21. Enriched GO terms in biological process.

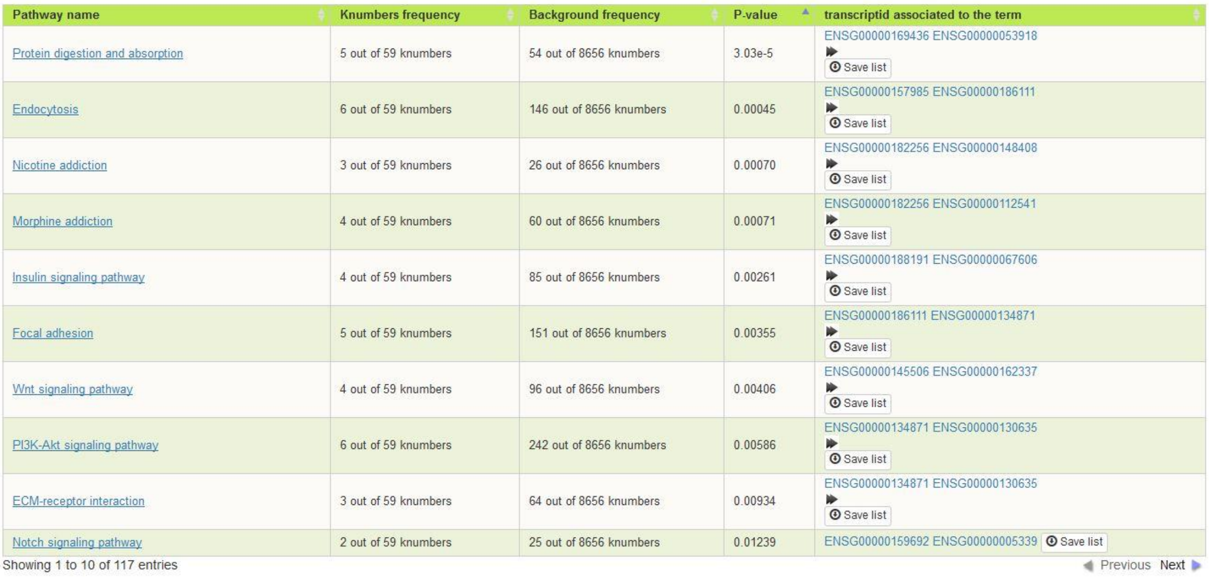

Pathway name	Knumbers frequency	Background frequency	P-value	transcriptid associated to the term
Protein digestion and absorption	5 out of 59 knumbers	54 out of 8656 knumbers	3.03e-5	ENSG0000169436 ENSG0000053918 Save list
Endocytosis	6 out of 59 knumbers	146 out of 8656 knumbers	0.00045	ENSG0000157985 ENSG0000186111 Save list
Nicotine addiction	3 out of 59 knumbers	26 out of 8656 knumbers	0.00070	ENSG0000182256 ENSG0000148408 Save list
Morphine addiction	4 out of 59 knumbers	60 out of 8656 knumbers	0.00071	ENSG0000182256 ENSG0000112541 Save list
Insulin signaling pathway	4 out of 59 knumbers	85 out of 8656 knumbers	0.00261	ENSG0000188191 ENSG0000067606 Save list
Focal adhesion	5 out of 59 knumbers	151 out of 8656 knumbers	0.00355	ENSG0000186111 ENSG0000134871 Save list
Wnt signaling pathway	4 out of 59 knumbers	96 out of 8656 knumbers	0.00406	ENSG0000145506 ENSG0000162337 Save list
PI3K-Akt signaling pathway	6 out of 59 knumbers	242 out of 8656 knumbers	0.00586	ENSG0000134871 ENSG0000130635 Save list
ECM-receptor interaction	3 out of 59 knumbers	64 out of 8656 knumbers	0.00934	ENSG0000134871 ENSG0000130635 Save list
Notch signaling pathway	2 out of 59 knumbers	25 out of 8656 knumbers	0.01239	ENSG0000159692 ENSG0000005339 Save list

Showing 1 to 10 of 117 entries Previous Next

Figure S22. Enriched KEGG pathways for the given gene sets.

2.4.2 Visualization Modules

Protein-Protein Interaction Network analysis

Here, we implemented a protein-protein interaction network (PPIN) viewer for integrating, visualizing, and analyzing gene list members in the protein network context in the system (Figure S23). This java plugin is based on Cytoscape.js [1], which supports network analysis and visualization. We use the BioGRID protein interaction data (version 3.4.159) to build the network topology. As a protein network graph, each node represents a protein, and each edge represents a known interaction between two nodes. We provide various network layout options and use the node size as a key

that visualizes the relative importance of each node with regard to the selected network topology measure. For example, degree centrality is a naive measure of the interacting neighbors of a node. Closeness and betweenness centrality are two of the most widely used global centrality measures. Furthermore, the option "Shortest path level" [2] on the function panel "**Layout"** allows the recruitment of extra neighboring nodes to build connections between any two nodes in the original input gene list.

The three major parts on the function panel of this PPIN viewer are:

- **Search**: Users can locate genes on the network subgraph by the gene symbol in the exact search. To find more than one protein in one query, users can separate the query with multiple gene symbols by commas.
- **Layout**: The protein network layout can be displayed in *Grid*, *Random*, *CoSE*, *Concentric*, *Breadthfirst*, *Arbor*, *Cola*, *Dagre*, and *Spread* manners. The network topology measures include degreeCentrality, degreeCentralityNormalized, closenessCentrality, closenessCentralityNormalized, and betweennessCentrality. In addition, the option "shortest path level" will expand the subnetwork to include connected paths that require only one or two extra stepping nodes (neighboring proteins) between any two nodes in the original input list. Recruited neighboring genes and edges for this expansion are discriminated in red.
- **Export:** The selected gene list or network can be exported as a Cytoscape JSON file, a text file of binary protein interactions, or an image in PNG or JPG format.

Table S1. The numbers of protein ID and non-redundant binary interactions for species.

	Protein IDs	Protein-Protein Interactions
Arabidopsis	10,216	48,374
Mouse	7,026	19,308
Human	17,515	320,510

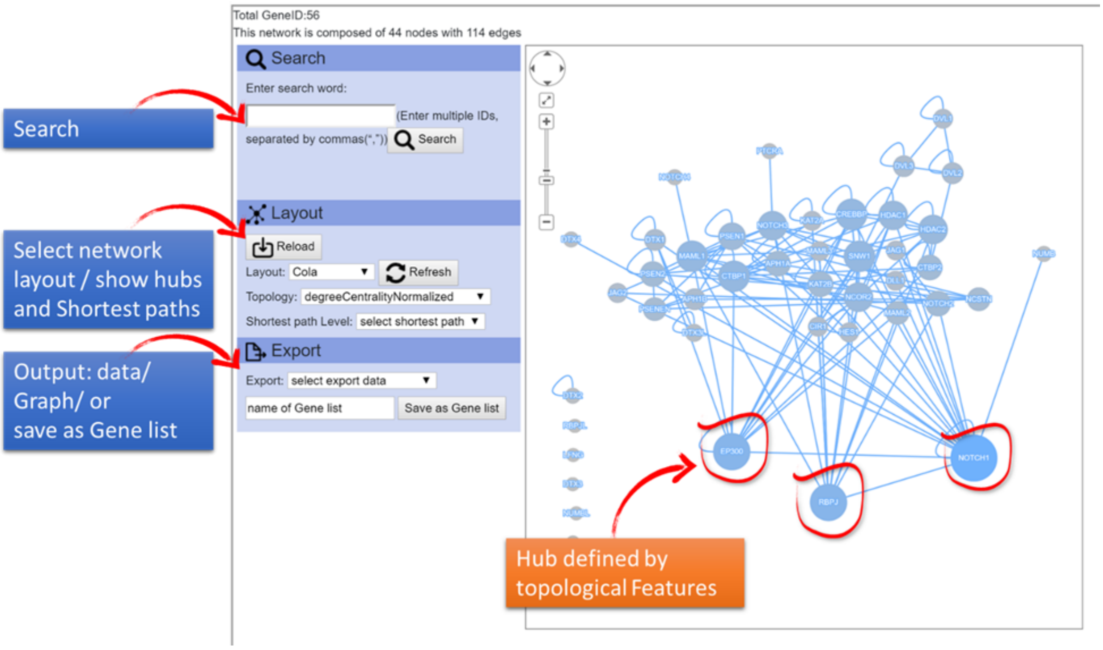

Figure S23. The protein interaction network viewer. Users can find and extract essential proteins via various layouts and methods.

Hierarchical Clustering Heatmap

The heatmap function is used to display a measurement from each sample with unsupervised hierarchical clustering in both samples and the selected gene lists. We integrated the interactive clustered heatmap visualization generated by Clustergrammer [3] into EpiMOLAS_web. The number of genes used to draw a heatmap is limited to 3000 because of the efficiency and rationality. The row annotations are the gene symbols with the dendrogram, while the column annotations are the samples with the dendrogram showing clusters between samples. We downloaded several human primordial germ cell WGBS datasets from this paper [4] for the following demonstration (Figure S24).

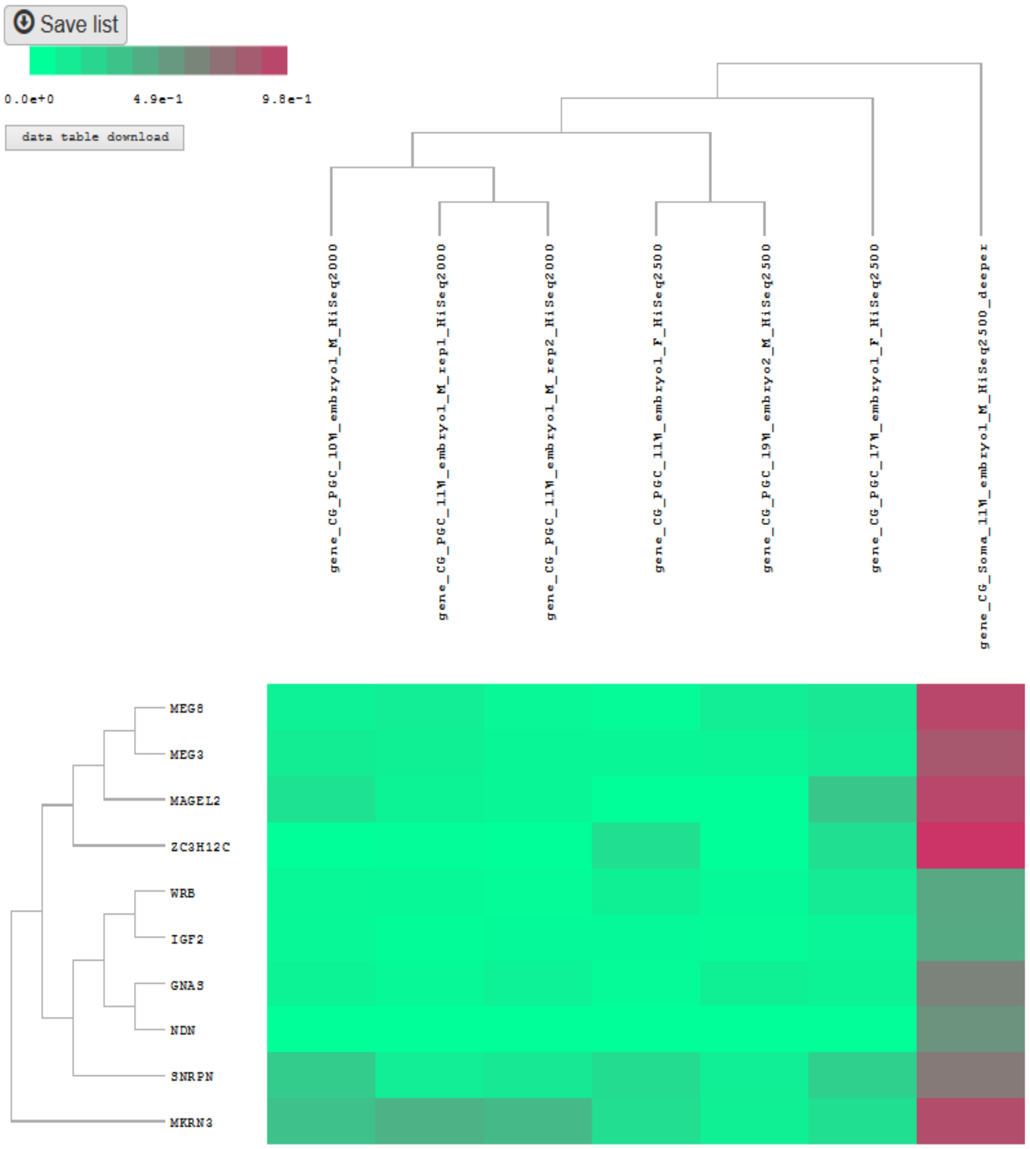

Figure S24. Hierarchical clustering heatmap. The row annotations are the gene symbols with the dendrogram, while the column annotations are the samples with the dendrogram showing clusters between samples. The green color represents hypomethylated genes and red color represents hypermethylated genes in the corresponding samples.

Venn diagram and Circos plot

Venn diagram is a graphical way to manipulate gene lists as sets. It is an interactive and intuitive visual way to find subsets that are either overlapping or exclusive to the gene sets of interest. It can produce a diagram to compare up to four gene sets (Figure S25). The Circos plot visualization module [5] is used to label gene locations (the chromosomal coordination) of all genes in the selected list(s). This circular genome data visualization supports a variety of plot types. In Figure S26, chromosomes are shown in the outermost circle, and the innermost circle shows the genomic coordinates of selected genes. The middle circle with a color gradient represents the density of the coding genes. Each bin size for counting the coding genes is 1 MB base pairs.

A

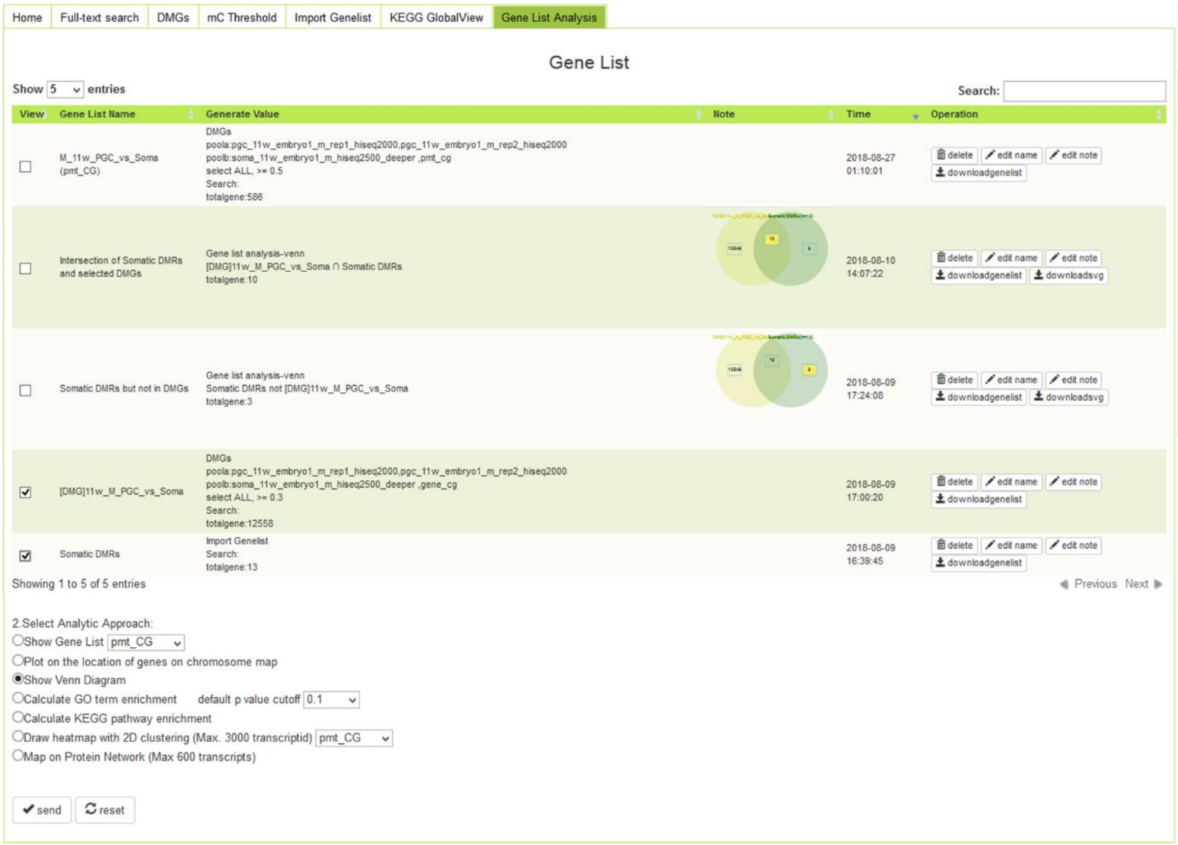

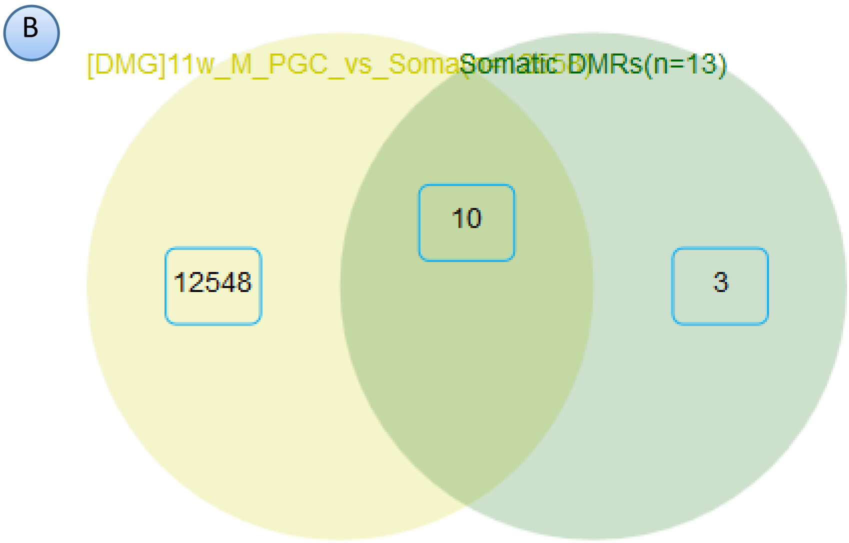

Figure S25. Venn diagram for two gene lists. (A) One is the differentially methylated genes (DMGs) that we select according to the criteria (Pool A: PGC_11W_embryo1_M_rep1_HiSeq2000, PGC_11W_embryo1_M_rep2_HiSeq2000 vs Pool B: Soma_11W_embryo1_M_HiSeq2500_deeper, Diff >= 0.3), 12558 genes are selected in this selection criteria. The other is the somatic DMRs mentioned in the paper (Guo, et al., 2015). (B) The intersection of literature-based somatic DMRs and the DMGs comprises 10 genes. The three genes that are not included in the selection occurred because of the null value on the gene body region of CG context in the mtables. The Venn diagram provides an efficient visualization interface for users to compare the collected or generated gene lists.

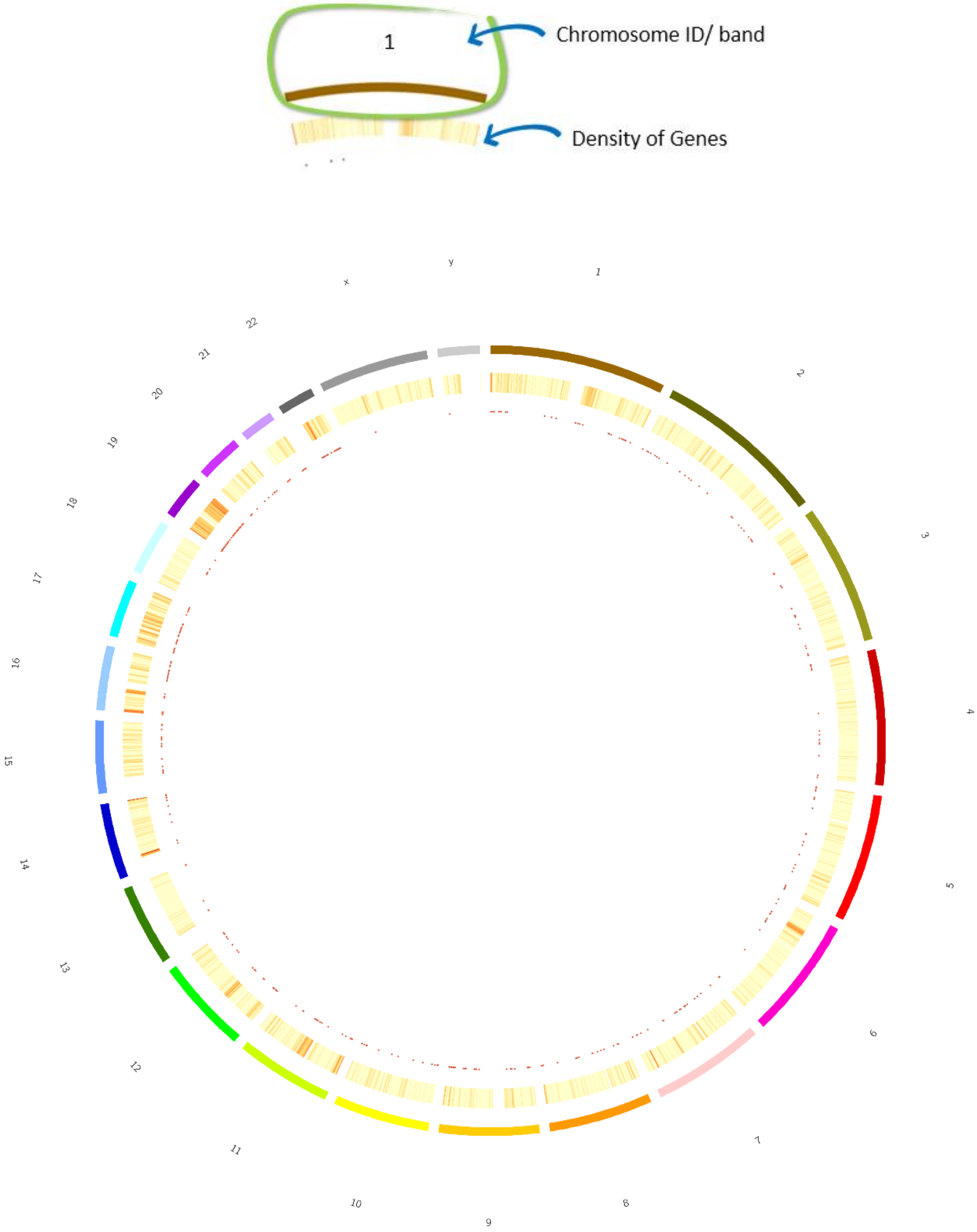

Figure S26. Circos plot of chromosomal location of a list of genes (gene list: M_11w_PGC_vs_Soma [pmt_CG]. The number of genes: 586). Chromosomes are shown in the outermost circle. The innermost circle shows the genomic coordinates of selected genes. The middle circle represents the density of coding genes with bin size 1 MB.

3. Reference

1. Franz M, Lopes CT, Huck G, Dong Y, Sumer O, Bader GD: **Cytoscape.js: a graph theory library for visualisation and analysis.** *Bioinformatics* 2016, **32:**309-311.
2. Lin CY, Chin CH, Wu HH, Chen SH, Ho CW, Ko MT: **Hubba: hub objects analyzer--a framework of interactome hubs identification for network biology.** *Nucleic Acids Res* 2008, **36:**W438-443.
3. Fernandez NF, Gundersen GW, Rahman A, Grimes ML, Rikova K, Hornbeck P, Ma'ayan A: **Clustergrammer, a web-based heatmap visualization and analysis tool for high-dimensional biological data.** *Sci Data* 2017, **4:**170151.
4. Guo F, Yan L, Guo H, Li L, Hu B, Zhao Y, Yong J, Hu Y, Wang X, Wei Y, et al: **The Transcriptome and DNA Methylome Landscapes of Human Primordial Germ Cells.** *Cell* 2015, **161:**1437-1452.
5. Krzywinski M, Schein J, Birol I, Connors J, Gascoyne R, Horsman D, Jones SJ, Marra MA: **Circos: an information aesthetic for comparative genomics.** *Genome Res* 2009, **19:**1639-1645.
